# *Streptomyces*-Derived Metabolites with Potential Photoprotective Properties—A Systematic Literature Review and Meta-Analysis on the Reported Chemodiversity

**DOI:** 10.3390/molecules25143221

**Published:** 2020-07-15

**Authors:** Jeysson Sánchez-Suárez, Ericsson Coy-Barrera, Luisa Villamil, Luis Díaz

**Affiliations:** 1 Doctoral Program of Biosciences, School of Engineering, Universidad de La Sabana, Chía 140013, Cundinamarca, Colombia; jeyssonsasu@unisabana.edu.co (J.S.-S.); luisa.villamil@unisabana.edu.co (L.V.); 2 Bioprospecting Research Group, School of Engineering, Universidad de La Sabana, Chía 140013, Cundinamarca, Colombia; 3 Bioorganic Chemistry Laboratory, Universidad Militar Nueva Granada, Bogotá 110111, Cajicá, Cundinamarca, Colombia; ericsson.coy@unimilitar.edu.co

**Keywords:** actinobacteria, *Streptomyces*, antioxidant activity, UV-absorbing, anti-inflammatory, photoprotection, sunscreen

## Abstract

Sun overexposure is associated with the development of diseases that primarily affect the skin, which can lead to skin cancer. Among the main measures of photoprotection is the use of sunscreens. However, there is currently concern about the reported harmful effects to both humans and the environment due to several of the sunscreen ingredients available on the market. For this reason, the search for and development of new agents with photoprotective properties is required. In searching for these metabolites, researchers have turned their attention to microbial sources, especially the microbiota in unusual hostile environments. Among the diverse microorganisms available in nature, Actinobacteria and specifically *Streptomyces*, have been shown to be a source of metabolites with various biological activities of interest, such as antimicrobial, antitumor and immunomodulator activities. Herein, we present the results of a systematic review of the literature in which *Streptomyces* isolates were studied as a source of compounds with photoprotective properties. A meta-analysis of the structure-property and structure-activity relationships of those metabolites identified in the qualitative analysis phase was also carried out. These findings indicate that *Streptomyces* are a source of metabolites with potential applications in the development of new, safe and more eco-friendly sunscreens.

## 1. Introduction

Actinic damage or injury in humans refers to the negative effects of solar ultraviolet radiation (UVR) and it is well-documented in skin biology [1]. UVR is the main etiological factor of several skin conditions, such as photoaging, melasma and skin cancers [2]. The latter condition is currently of great concern due to the increase in the incidence rate [3]. On the other hand, photoprotection is defined as the set of chemical, physical and behavioral-based actions to prevent or counteract the actinic damage [1]. Likewise, among the different preventive photoprotection measures, the use of sunscreens is one of the main recommended actions to avoid the harmful effects of sun exposure, after clothing and behavior strategies [3].

A sunscreen works by mitigating the deleterious effects of UVR on skin. Therefore, sunscreens have compounds that can absorb, reflect or scatter UVR [4]. These agents are known as UV filters and are classified according to their chemical nature into inorganics and organics. The former consists of titanium dioxide (TiO_2_) and zinc oxide (ZnO) and the latter consists of different options depending on the regulatory norms in each country (e.g., in the FDA monograph there are 16 organic UV filters) [5,6]. Regardless of the type of UV filter, it has been shown that both can act mainly by absorbing UVR [7]. In the case of organic UV filters, the occurrence of conjugated double bonds is a crucial feature to being able to absorb UV light, which is then dissipated as heat [4]. However, sunscreening agents are currently associated with some adverse effects to a greater or lesser extent, either by affecting human health or by polluting natural ecosystems or both, in the worst cases [8,9,10,11].

Several side effects have been associated with some ingredients in sunscreens currently available in the market. For example, several studies have found that some of the most used UV-filters (e.g., benzophenone-3,3-benzylidene camphor, 3-(4-methyl-benzylidene) camphor, 2-ethylhexyl 4-methoxy cinnamate, homosalate, 2-ethylhexyl 4-dimethylaminobenzoate and 4-aminobenzoic acid, PABA) can act as endocrine-disrupting chemicals [12]. In addition, camphor and benzophenone derived UV-filters have shown to be detrimental agents against marine organisms [13]. There is some reported information stating that these compounds can be bioaccumulated in marine organisms that are part of the food chain [14] and some are associated with harmful effects on coral health [15,16].

Furthermore, although sunscreen production has focused on the use of compounds that affect the transmission of UVR to the skin (both inorganic and organic UV filters), it is known that a photoprotective effect could be achieved through the modification of the UVR physiological effects on the skin [17,18]. Particularly, among the claims made for current sun protection products, some state that these products must incorporate antioxidant and anti-inflammatory activities in addition to the UV-absorbing/filtering abilities [19,20,21]. This upgrade is pertinent, since the actinic damage involves both oxidative stress and inflammatory response in the skin [22]. In fact, bearing in mind that the generation of reactive oxygen species (ROS) and inflammation are early steps in cutaneous photocarcinogenesis [23], sunscreens might become skin cancer chemopreventive products by the incorporation of compounds that exhibit antioxidant and anti-inflammatory activities [24].

Considering the abovementioned facts, there is not an ideal sunscreen owing to the described limitations for the currently available sun protection products. Therefore, there is an undeniable need to find safer, eco-friendly and upgraded alternatives to serve as new ingredients for sunscreen improvement. In this regard, natural products constitute an essential opportunity to discover compounds with engaging biological activities for both pharmaceutical and cosmetic industries [25,26]. Despite such promising chemical and biological traits, research on compounds of natural origin has several significant logistical and technological disadvantages that limit their industrial application, such as the naturally occurring complex structures (expensive and time-consuming to synthesize) [27], low yield during the isolation process and limited availability of source organisms to yield higher amounts to satisfy supply [28,29]. However, the technological advances in biotechnology and analytics have identified improved approaches to overcome such challenges and produce compounds of industrial interest on a commercially large-scale based on natural molecules [30,31]. In this context, the success of microbial biotechnology for the industrial application of products derived from nature is well-known. For instance, several microorganism-derived products, such as antibiotics, enzymes, enzyme inhibitors, peptides and vaccines, are currently used in the pharmaceutical industry [32]. An advantage of the use of microorganisms as biofactories of biologically active compounds lies in their fast-growing attribute [32,33]. Another convenient feature is the possibility to produce the target compounds by fermentation, which is relatively easier than obtaining them from a macroorganism [32]. In addition to these facts, the diversity of microorganisms, compared to macroorganisms, is staggeringly higher. This diversity may be extended to the availability of a broad repertoire of different biosynthetic gene clusters [34,35,36], which can provide a varied and broad number of compounds with unique chemical structures [37,38].

Within the vast biodiversity of microorganisms available in nature, members of actinobacterial genus *Streptomyces* are one of the most prolific and successful groups of bacteria producers of bioactive compounds [39]. It is estimated that they are major producers of antibiotics used in human infection treatments [40,41]; approximately 70% of clinically used antibiotics come from actinomycetes, mainly *Streptomyces* [42]. Moreover, other activities, such as antitumor, immunomodulator and hypercholesteremic activities, have been reported for *Streptomyces*-derived metabolites, even the production of enzymes with therapeutic value [39]. In the specific instance of photoprotective activities, several actinobacterial species have also been shown to produce metabolites with antioxidant and UV-absorbing capabilities [43,44,45,46]. However, the potential of *Streptomyces* strains as a source of metabolites with the capability to be applied as photoprotective agents to be included into sunscreening products have been poorly explored.

*Streptomyces* have a unique genetic structure comprising complex regulatory networks. Such a structure involves different gene clusters, even silent/cryptic ones, encoding several metabolic pathways for the synthesis of functionally and structurally different secondary (also called specialized) metabolites [47]. Consequently, *Streptomyces*-related microorganisms can produce very interesting metabolites and several of them have bioactivities and applications of industrial interest. This chemical repertoire has conferred particular biological traits to *Streptomyces* to adapt to different environmental conditions [48]. Therefore, they can be found on air, soil, freshwater and seawater [39] and not only as free-living organisms but also as symbionts [49]. This ubiquity suggests the evolutionary success of their secondary metabolite arsenal and correlates with the relevance of their ecological functions [50,51]. Furthermore, given that the bioactivity profile of *Streptomyces*-derived metabolites is not yet fully described, we intended to explore what metabolites may be associated with a photoprotective role. With this in mind, we accomplished a systematic literature review of metabolites produced by *Streptomyces* spp. that have UV-absorbing, antioxidant and anti-inflammatory activities—these activities can contribute to achieve a photoprotective action by mitigating the deleterious effects of UVR [18]. Subsequently, from the group of compiled compounds during the systematic survey, we classified the compound library into several subclasses regarding the type of secondary metabolite. Additionally, we explored their potential as photoprotective agents through a meta-analysis of the structure-property (SPR) and structure-activity relationship analysis (SAR). This systematic review and meta-analysis aimed to identify, summarize and evaluate the evidence regarding the potential of *Streptomyces* strains as a biological source of metabolites with photoprotective-related properties.

## 2. Results

### 2.1. General Findings

**Figure 1:**
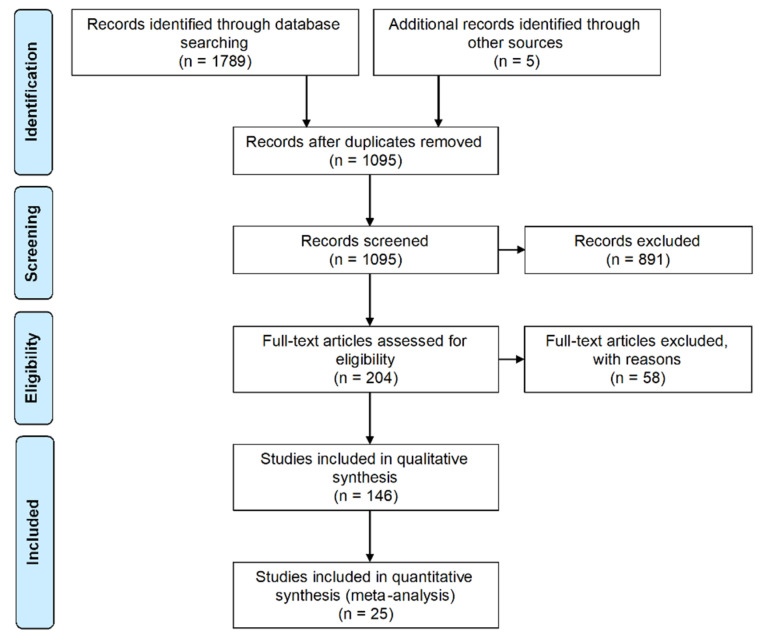
PRISMA flow diagram. Flowchart of systematic literature search according to PRISMA guidelines. Modified from: Moher D, Liberati A, Tetzlaff J, Altman DG, The PRISMA Group (2009). Preferred Reporting Items for Systematic Reviews and Meta-Analyses: The PRISMA Statement. PLoS Med 6 (7): e1000097 [52].

The literature search identified 1789 potential studies. From this number, 694 articles were found to be duplicates and the remaining 1095 studies were screened by reading the titles and abstracts according to the inclusion/exclusion criteria. From this screening, 204 papers were selected for full-text assessment. Accordingly, 146 papers were finally included for data extraction. As most articles reported antioxidant capacity under the DPPH (2,2-diphenyl-1-picrylhydrazyl) radical scavenging assay, a meta-analysis approach was additionally feasible to compare results among them. In this regard, we identified 23 studies providing the half-maximal inhibitory concentration (IC_50_, that is, concentration required for 50% reduction of the DPPH radical) as a result of DPPH assays. Furthermore, from the reported datasets of two articles, we were able to calculate the respective IC_50_ values. Thus, 25 articles were included in the meta-analysis. Figure 1 summarizes the full process.

**Figure 2:**
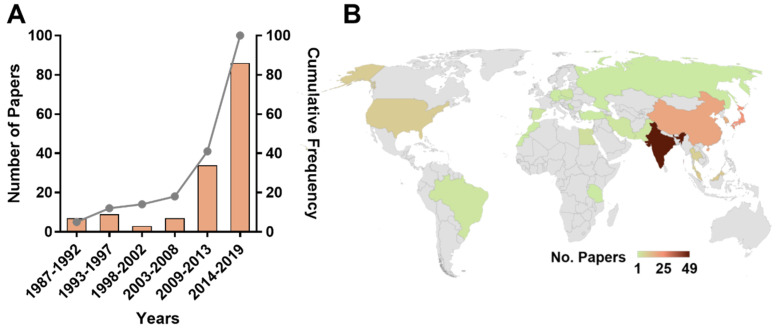
The number of papers in the literature by years and country of origin. (**A**) Publication distribution over the time. The first *y*-axis shows the number of papers and the second *y*-axis shows the cumulative frequency distribution. (**B**) World map showing the countries where the articles included in this review were produced, specifically, the corresponding author affiliation. Only in nine articles (6.2%), the sampling place for isolating *Streptomyces* was different from the country of the corresponding author (See Figure S1).

Although the earliest report found in the review dates back 1987, the greatest number of articles (59.0%) were published between 2014 and 2019 (Figure 2A). This increased number of publications was associated with the number of participating countries. Until 2008, publications were restricted to five countries; in the period 2009–2013, they increased to eight and during 2014–2019 they reached 18, resulting in a total of 23 countries (Figure 2). However, it should be noted that the publication number is still insignificant and regions such as Latin America, Africa and most of Europe are poorly explored territories in this research field (Figure 2B).

**Figure 3:**
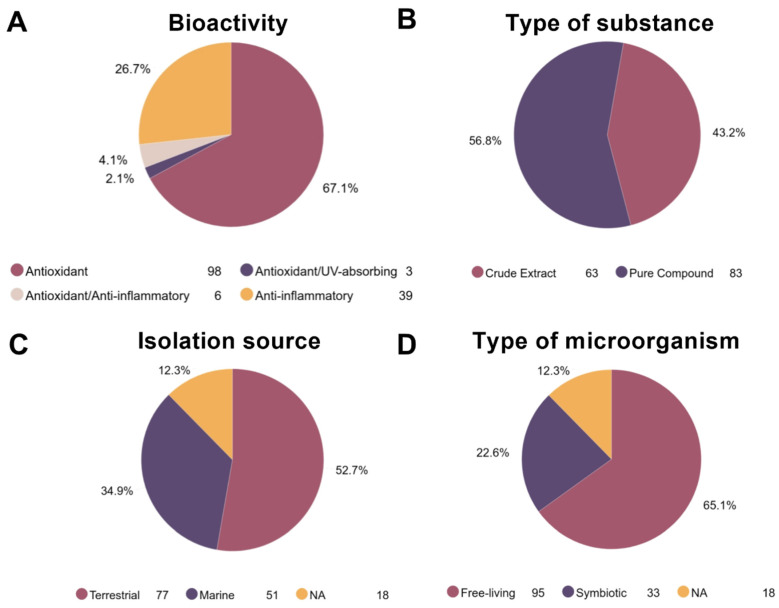
Characteristics of the information registered in the selected articles. The publications were grouped according to the information extracted for the analysis. (**A**) Percentage of articles by biological activity. (**B**) Percentage of articles that evaluated either crude extracts or pure compounds. (**C**) Percentage of articles by the environment where the isolation occurred (i.e., terrestrial, marine or not available). (**D**) Percentage of articles by the type of *Streptomyces* strain isolated (i.e., free-living, symbiotic or not available). At the bottom of each pie chart, the number of papers for each group is presented.

Regarding biological activities, most of the articles (73.3%) focused on antioxidant capacity (Figure 3A); 67.1% evaluated only the antioxidant potential, whereas 6.2% involved measurement of the antioxidant capacity along with other bioactivities (i.e., anti-inflammatory or UV-absorbing). The anti-inflammatory potential was reported in 26.7% of the studies. The UV-absorbing ability was evaluated in only three papers, representing 2.1% of the selected publications. In relation to the type of substance, there was no marked difference between crude extracts and pure compounds. Crude extracts were used by 43.2% of the articles and pure compounds were used by the remaining 56.8% (Figure 3B). Considering that *Streptomyces* are ubiquitous in nature, either in terrestrial or marine environments [53,54], we also examined the habitat source where the *Streptomyces* strain was isolated (Figure 3C). Although some papers (12.3%) did not document the origin of the isolated strain, most strains (52.7%) were isolated from terrestrial environments and 34.9% of strains were isolated from marine habitats. Finally, the role of symbiosis in the evolution of *Streptomyces* is a very important factor, particularly in their gene machinery for the biosynthesis of secondary metabolites [49]. For this reason, we considered whether the strains were isolated as free-living or symbiotic microorganisms (Figure 3D). We found that most studies focused on free-living *Streptomyces* (65.1%) and very few studies used symbiotic strains (22.6%).

### 2.2. Streptomyces as a Biological Source of Photoprotective Metabolites

As mentioned above, photoprotection can be achieved by compounds participating as UV-absorber, antioxidant and anti-inflammatory agents [19,20,21]. Actually, a better photoprotective effect is reached by the combination of those compounds exhibiting these activities [55,56]. Hence, to evaluate the potentiality of *Streptomyces* as a source of photoprotective metabolites, we explored the literature records on *Streptomyces* strains with any of these activities (i.e., UV-absorbing, antioxidant and anti-inflammatory). The crude extract activity is a valuable measure of an organism’s potential to produce metabolites with a particularly interesting function. In this case, 63 studies were found to evaluate the bioactivity of crude extracts obtained from *Streptomyces* strains. Table 1 summarizes the number of strains, isolation source and type of microorganism, along with the evaluated bioactivity.

**Figure 4:**
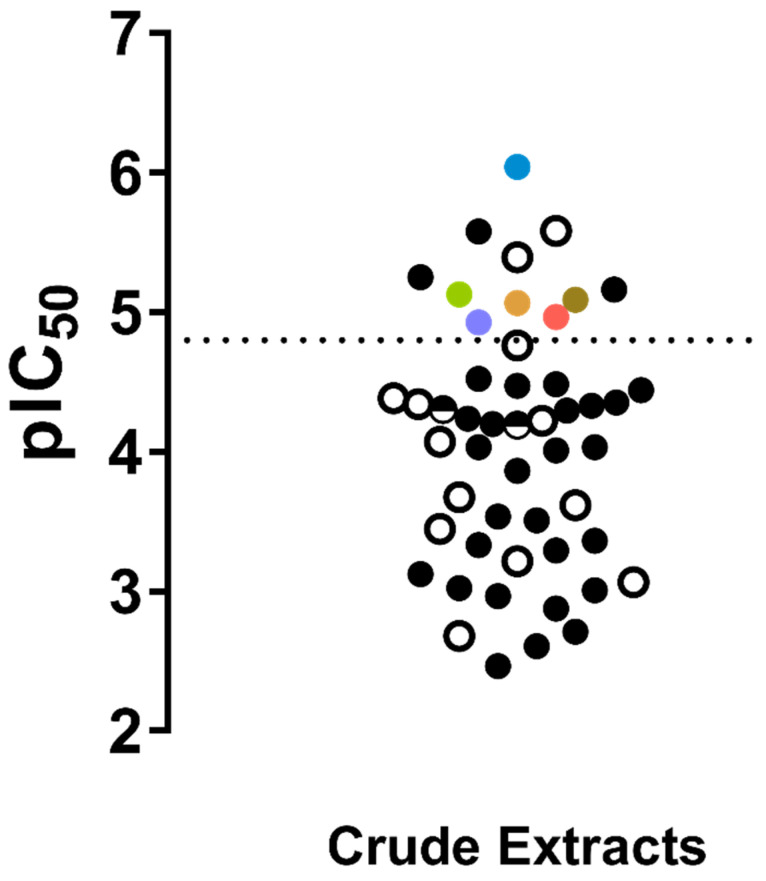
Distribution of antioxidant capacity of *Streptomyces*-derived crude extracts measured by the DPPH assay. The IC_50_ are presented as −logIC_50_ (g mL^−1^). The empty circles (○) represent crude extracts of marine origin and filled circles (●) represent crude extracts of terrestrial origin. The colored circles show reference antioxidant compounds (i.e., ascorbic acid ●, butylhydroxytoluene ●, α-tocopherol ●, rutin ●, novobiocin ●, quercetin ●). Dotted line indicates the minimum limit potency of reference antioxidant compounds.

The antioxidant capacity was evaluated in 88 of the 89 extracts studied. As expected, 91.0% (n = 81) of the publications reported DPPH as the selected method to determine the antioxidant potential, thus facilitating the comparison of the results among studies. The IC_50_ of the *Streptomyces* crude extracts were obtained and listed in Table 2 and converted to a logarithmic scale and presented in a dot blot (Figure 4). It was found that some crude extracts had radical scavenging capacity at a level similar to that of some reference antioxidant compounds that was independent of the source ecosystems (i.e., marine or terrestrial).

On the other hand, the biological activity evaluation of pure compounds could lead to more accurate analysis because the plausible mechanisms of action and structure-activity/property relationship analysis could be proposed. Both have a priceless value for the development of products in the pharmaceutical industry [120]. In this sense, 75 *Streptomyces* strains were identified and from these, 133 biologically active purified compounds were reported (Table 3; see Table S2 for detailed data). Interestingly, it was found that some *Streptomyces* strains produce more than one bioactive compound. Unlike crude extracts, we could find compounds where the UV-absorbing activity was evaluated and the number of anti-inflammatory activity reports was significantly higher (i.e., 66 anti-inflammatory compounds—see Table 3—versus six anti-inflammatory extracts—see Table 1).

### 2.3. Chemical Space Analysis

To explore the ‘biologically relevant chemical space’—in terms of photoprotective compounds – reported so far, the metabolites registered in those articles included in the qualitative analysis phase were extracted to build a custom-made compound library. For this, their chemical structures were sketched and converted into SMILES notation and from this format, the chemical space analysis was performed using the software Osiris DataWarrior v5.2.1 (Idorsia Pharmaceuticals Ltd., Switzerland) [203]. In the Table S2, the identification number given for each compound is presented along with its respective SMILES notation.

As stated in the Section 1, our primary goal is to explore the evidence about the potential of *Streptomyces* as a producer of photoprotective metabolites. In this sense, we aimed to characterize the biological profile and chemical diversity of the *Streptomyces*-derived metabolites (herein identified and compiled) through a chemoinformatics-based analysis. Such an analysis was accomplished using the software DataWarrior involving three steps—(i) selection of the descriptor for the compound clustering, (ii) similarity analysis of the compounds, which is a useful approach to filter compounds and (iii) Structure-Property Relationships (SPR) and Structure-Activity Relationships (SAR) analyses of the custom-made compound library, whereby the correlation between chemical structures and bioactivity is established.

**Figure 5:**
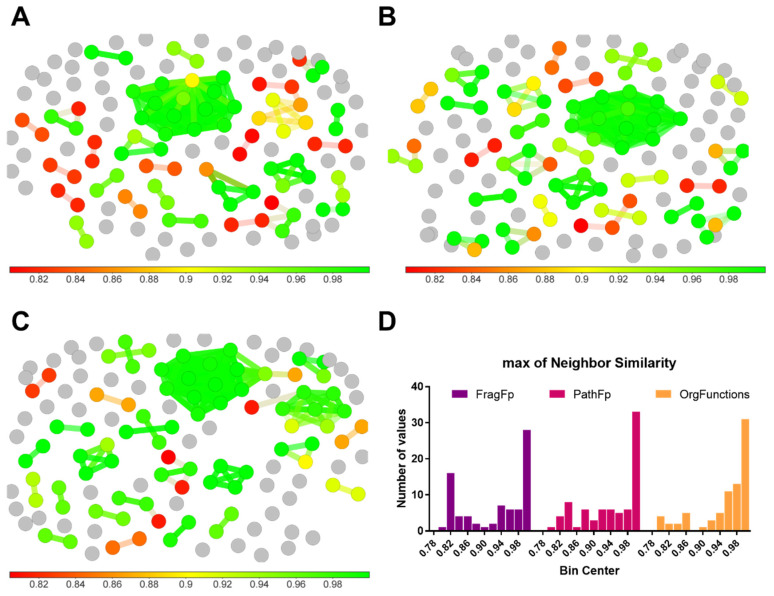
Similarity analysis of the chemical space using different descriptors. The level of similarity is indicated by a color scale bar (located at the bottom of each dot plot), from red (similarity = 0.8) to green (similarity = 1.0). Similarity analysis of the compound library was performed using (**A**) *FragFp* descriptor, (**B**) *PathFp* descriptor and (**C**) *OrgFunctions* descriptor. (**D**) Distribution frequency of each similarity value calculated by each descriptor.

DataWarrior calculates similarity among molecules using descriptors that are obtained from particular structural features of test compounds [203]. To define the best descriptor to be used for the subsequent analysis, we evaluated the performance of some descriptors in the similarity analysis of the compound library. Hence, we evaluated the following three descriptors—(a) *FragFp* (a substructure fragment dictionary based binary fingerprint) [203], (b) *PathFp* (encodes all distinct linear strands of up to seven atoms within a molecule) [203] and (c) *OrgFunctions* (perceives molecules from a synthetic chemistry’s point of view, that is, it classifies organic functional groups focusing on steric and electronic features) [203]. Accordingly, the SMILES-based format of compounds was first incorporated as a datasheet into DataWarrior. A similarity analysis (with default settings) using each descriptor (i.e., *FragFp*, *PathFp* and *OrgFunctions*) was then executed as shown in Figure 5. We found that *OrgFunctions* was the descriptor that arranged the compound set by higher similarity values. Thus, the proportions of compounds with similarity higher than 0.9 were 65%, 73% and 83%, with *FragFp*, *PathFp* and *OrgFunctions*, respectively (see Figure 5D). In consequence, the similarity analysis of the metabolites was done using the *OrgFunctions* descriptor and the analysis with all the compounds saved in our library (133) was carried out as shown in Figure 5C.

A similarity analysis allows us to organize and measure the level of chemical diversity within the chemical space defined by the characteristics of the compound group [204]. In this particular aspect, such a measure of chemical diversity may be used as an indicator of biosynthetic aspects, for example, the heterogeneity of the biosynthetic pathways employed by a biological organism or group of them (e.g., organisms of the same species or genus) to produce a given group of compounds [204,205].

**Figure 6:**
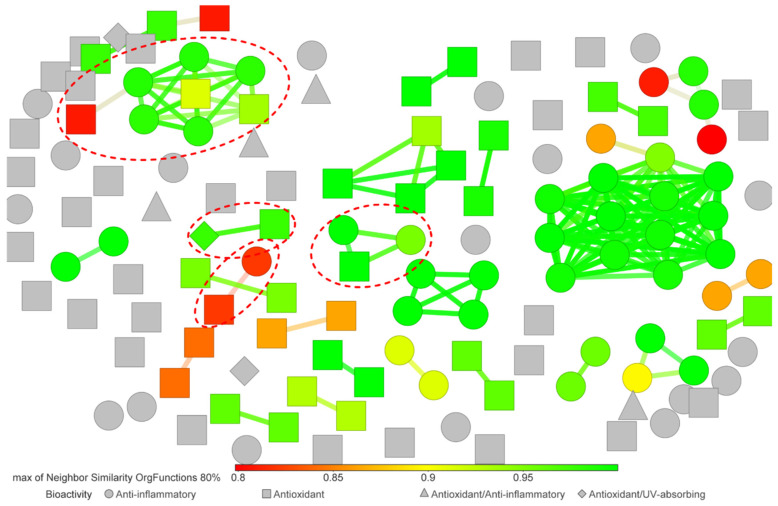
Similarity analysis of *Streptomyces*-derived photoprotective compound library. The similarity analysis was carried out using the *OrgFunctions* descriptor. The color scale bar indicates the level of similarity from red (0.8) to green (1.0). Each marker shape indicates the bioactivity reported for a given compound. The red dotted circles highlight compound clusters where metabolites with different bioactivity were grouped together.

The level of chemical diversity and possible grouping of the compounds based on the biological activity was assessed. As shown in Figure 6, several unique fingerprints were observed, supporting a great diversity in the custom-made library. In addition, several clusters were identified that included different numbers of compounds. Particularly, some compounds (40%) were not able to be clustered, since the descriptor used did not find shared structural characteristics at a comparatively significant level. Thus, the analysis of anti-inflammatory and antioxidant compounds was carried out separately. It is important to point out that since the compounds **60**, **64**, **65** and **66** exhibited both bioactivities, they were included in the analysis of each group. The groups were defined as (a) anti-inflammatory metabolites, with 66 compounds and (b) antioxidant metabolites, with 71 compounds.

**Figure 7:**
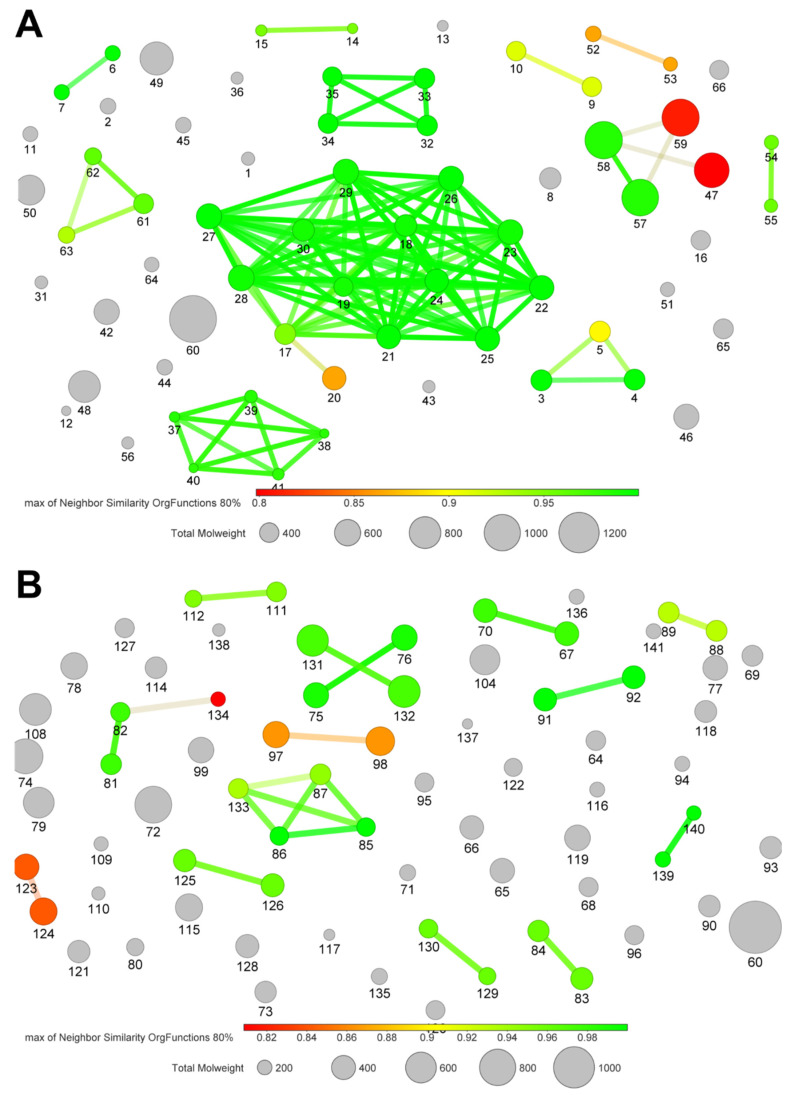
Similarity analysis of anti-inflammatory and antioxidant *Streptomyces*-derived metabolites. The similarity analysis was performed using the *OrgFunctions* descriptor. The color scale bar indicates the level of similarity from red (0.8) to green (1.0). Each marker size indicates molecular weight (g mol^−1^). (**A**) Compounds with anti-inflammatory activity. (**B**) Compounds with antioxidant capacity.

Fewer unique fingerprints were found in the anti-inflammatory compounds’ subgroup (35) in comparison to the antioxidant subgroup (55) (Figure 7A,B). In both subgroups, there was a wide range in molecular weight. As expected, clustering of structurally related compounds also grouped compounds with quite similar size, since other atomic arrangements not only affect the molecular weight but also the similarity, resulting in lower values. These facts could be related with the high diversity of biosynthetic pathways employed by *Streptomyces* to produce these compounds. To evaluate this possibility, the next task was to cluster the compounds of the evaluated subgroups according to their biosynthesis pathway (i.e., alkaloids, amides, phenylprenoids, polyketides, flavonoids and terpenoids).

**Figure 8:**
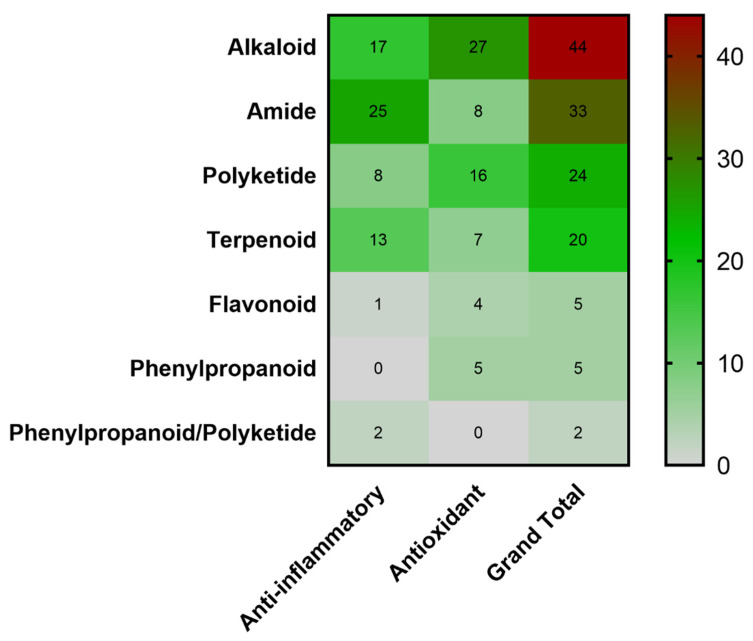
Heatmap analysis of *Streptomyces*-derived compound library. The compounds were classified as alkaloids, amides, polyketides, terpenoids, flavonoids, phenylpropanoids and mixed phenylpropanoid/polyketide according to the biosynthetically based chemical structure. Inside each cell, the number of compounds in the given group is presented. The color bar represents the number of compounds, gray (minor values), green (middle values) and red (high values).

Each metabolite in the compound library was classified as alkaloids, amides, phenylpropanoids, polyketides, flavonoids and terpenoids based on the elemental units identified in the molecular structure. Since compounds **52** and **53** showed mixed elemental units between phenylpropanoid and polyketide, it was necessary to incorporate a mixed phenylpropanoid/polyketide group. As shown in Figure 8, our compound library was mainly represented by alkaloids (33.1%) and amides (24.8%). Despite several of these compounds exhibiting mixed building blocks, they were classified into those classes due to the diversification/evolutionary concept that nitrogen-containing metabolites can imply specialized metabolism [206]. Interestingly, a singular trend was observed within the custom-made dataset, since the anti-inflammatory subgroup was mainly composed by amides, while the antioxidant subgroup was mainly represented by alkaloids. In general, it is important to clarify that *Streptomyces* has different biosynthetic pathways to produce metabolites with these biological activities.

Further analyses were also performed to establish molecular characteristics that may be associated with a specific biological activity. Particularly, a structure-property analysis (SPR) and structure-activity analysis (SAR) was performed with each subgroup of compounds, anti-inflammatory and antioxidant, respectively.

### 2.4. SPR and SAR Analysis

Owing to the anti-inflammatory activity was evaluated using different approaches, there was not a method used with a significant frequency to be selected. For instance, edema inhibition was reported in six papers. However, the results were not strictly comparable, since some evaluations were done on mice while the others involved rats as a model. Furthermore, some edema evaluations were induced in paw and others in the ears. Other remarkable facts were the variety in the methods to quantify inflammatory mediators—for example, ELISA, qPCR, Flow cytometry, Luminex 100 system. Even the target analyte varied mostly through the articles—for example, iNOS, COX-1, COX-2, TNF-α, IL-1β, -2, -4, -5, -10, -13. Therefore, the differences in the anti-inflammatory activity potency among metabolites could not be evaluated. Conversely, an SPR analysis was carried out in this case to visualize plausible relationships between structure and physicochemical characteristics. In contrast, most of the antioxidant capacity was evaluated using the DPPH radical scavenging assay, allowing the SAR analysis for these compounds.

Several Food and Drug Administration (FDA)-approved UV filters have shown anti-inflammatory activity [207]; it is even discussed that this anti-inflammatory activity promotes the photoprotective action of these UV filters [208]. In the case of SPR analysis, we included the UV filters with anti-inflammatory activity reported by Couteau et al. [207] as reference standards. The chemical structure was translated into SMILES annotation and analyzed using DataWarrior (i.e., 4-methylbenzylidene camphor, Benzophenone-3, Benzophenone-5, Bis-Ethylhexyloxyphenol methoxyphenyl triazine, Butylmethoxydibenzoylmethane, Diethylamino hydroxybenzoyl hexyl benzoate, Diethylhexylbutamidotriazone, Disodium phenyl dibenzimidazole tetrasulfonate, Ethyl hexyl methoxycinnamate, Ethylhexylsalicylate, Homosalate, Isoamyl p-methoxycinnamate, Methylene bis-benzotriazolyl tetramethylbutylphenol, Octocrylene, Octyl triazone, Octyldimethyl PABA, PEG-25 PABA and Phenylbenzimidazole sulfonic acid).

**Figure 9:**
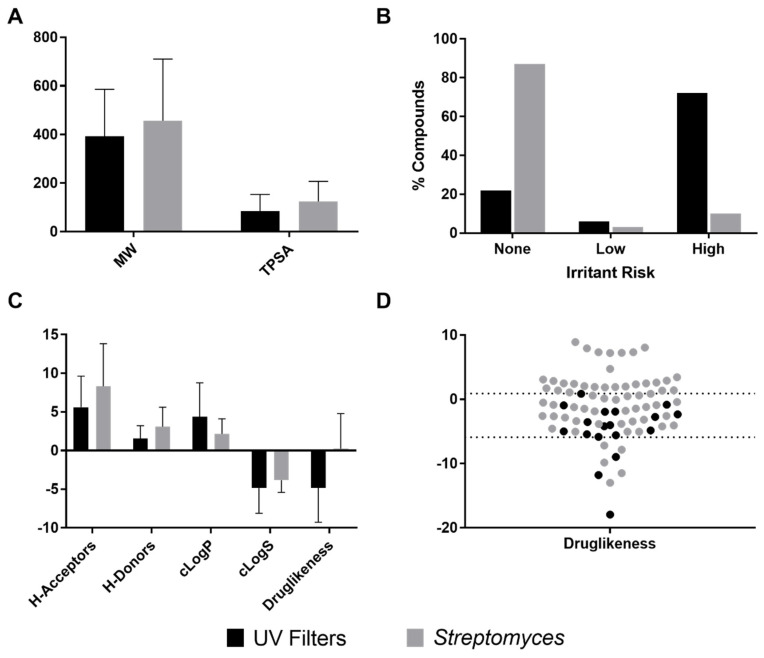
Structure-Property Relationships (SPR) analysis of *Streptomyces*-derived anti-inflammatory compounds. Several physicochemical properties of the anti-inflammatory metabolites were calculated theoretically by DataWarrior. Data in black represent values of UV filters and data in gray represent values of *Streptomyces*-derived metabolites. (**A**) Molecular weight (MW) and topological polar surface area (TPSA). (**B**) Irritant risk indicator, calculated as none, low and high risk. (**C**) *H*-Acceptors, *H*-Donors, cLogP, cLogS and druglikeness. (**D**) Dot plot of druglikeness values.

Considering that physicochemical properties, such as partition coefficient, solubility and molar mass are important properties of active agents affecting skin penetration [209], we calculated parameters associated with these properties (i.e., molecular weight, topological polar surface area (TPSA), octanol/water partition coefficient (cLogP), aqueous solubility (cLogS)), along with *H*-acceptors, *H*-donors and drug likeness, which are valuable criteria in pharmaceutical agents’ discovery [210,211]. Additionally, we calculated the irritant risk, which is a common side effect in sunscreen ingredients [212]. Figure 9 shows that irritant risk (Figure 9B) and druglikeness values (Figure 9C) were the properties with the most substantial differences. To better understand druglikeness differences, a dot plot was generated including UV filters versus *Streptomyces*-derived anti-inflammatory metabolites. It was found that most UV filters (83.3%) exhibited a druglikeness among −6 y 1, as well as many anti-inflammatory compounds (50.8%), as shown in Figure 9D. Regarding the risk of a compound to be an irritant, it was found that most UV filters (72%) were classified as high risk, as previously reported [212], in opposition with *Streptomyces*-derived anti-inflammatory compounds, in which only 10% had a high risk of being irritant (Figure 9B). This result gives an idea of the possibility of finding safer compounds in *Streptomyces*-derived metabolites for the development of better sunscreens.

Regarding the antioxidant subgroup (n = 71), 39 compounds where evaluated using the DPPH assay; 30 compounds reported the IC_50_ and it was also possible to calculate the IC_50_ of 4 other compounds using the data available in the publications (Table 4, compounds **64**, **65**, **66** and **69**). Additionally, 11 compounds that, although evaluated by the DPPH radical scavenging assay, were not active or did not achieve more than 50% inhibition at the highest concentration evaluated were also incorporated (i.e., **61**, **62**, **63**, **100**, **101**, **102**, **103**, **105**, **106**, **107** and **113**). This information was included with the aim to promote a more robust analysis and allow the identification of activity cliffs (pairs of structurally similar compounds that have a large potency difference [213]). In total, including active and non-active agents, 45 compounds were involved in the SAR analysis.

**Figure 10:**
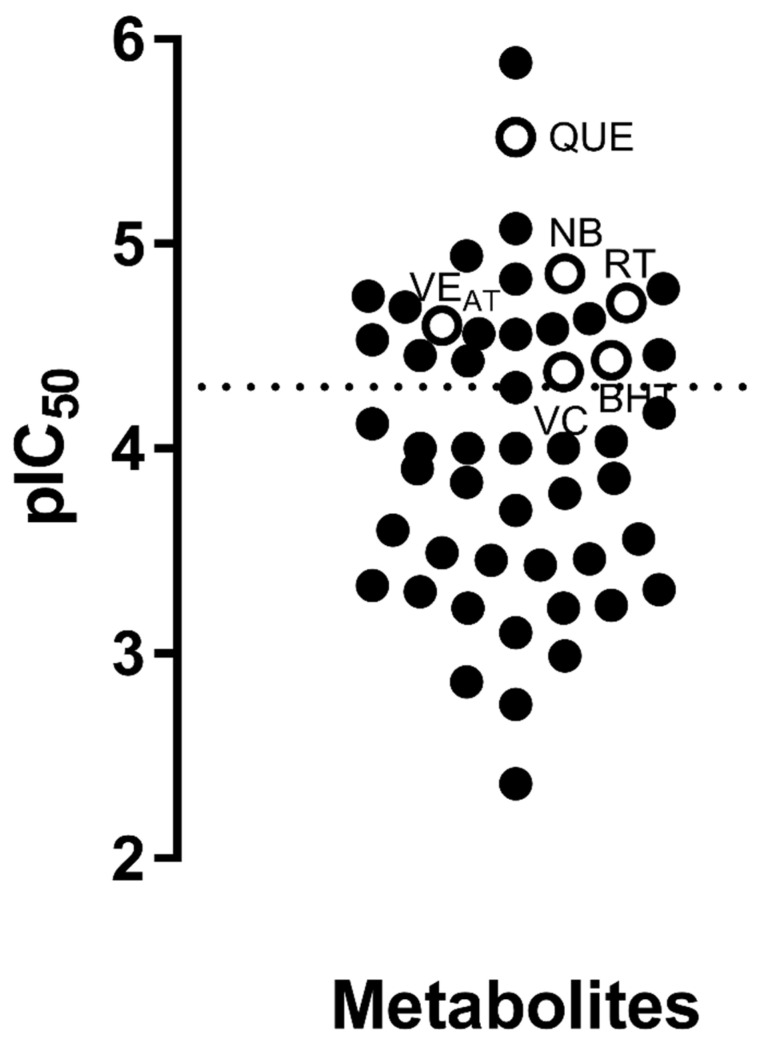
Distribution of *Streptomyces*-derived metabolites with DPPH radical scavenging capacity. The pIC_50_ of each compound was plotted along with some reference antioxidant compounds. Each black dot indicates a *Streptomyces*-derived metabolite and each empty dot indicates a reference antioxidant compound. VC, vitamin C; BHT, butylated hydroxytoluene; VE_AT_, vitamin E (α- tocopherol); NB, novobiocin; and QUE, quercetin. Dotted line indicates the minimum limit potency of reference antioxidant compounds.

Once establishing the compounds that satisfied the requirements for the SAR analysis, the IC_50_s data were standardized, first in molarity units (M) and then expressed logarithmically as −logIC_50_ (M) (Table 4). Figure 10 presents the IC_50_ values of the *Streptomyces*-derived metabolites along with antioxidant reference compounds such as vitamin C (VC), butylated hydroxytoluene (BHT), vitamin E (as α- tocopherol, VE_AT_), novobiocin (NB) and quercetin (QUE), in order to illustrate the potency of the antioxidant capacity of the *Streptomyces*-derived compounds.

There was a wide diversity in the potency of the free-radical scavenging capacity, since the IC_50_s varied from 1.3 (**104**) to 4330 μM (**118**). To evaluate the implications of the chemical structure in the heterogeneity of the antioxidant potency, a similarity analysis was conducted (using the *OrgFunctions* descriptor in the same way as with the group of anti-inflammatory compounds). The similarity analysis indicated the existence of nine clusters and with these in hand, a Structure-Activity Landscape Index (SALI) analysis was performed. A SALI analysis allowed us to identify and quantify activity cliffs, and, in this way, provided details of the structural key features that contribute to the antioxidant capacity [215]. Table 5 shows the results of the 17 compound pairs found by DataWarrior.

**Figure 11:**
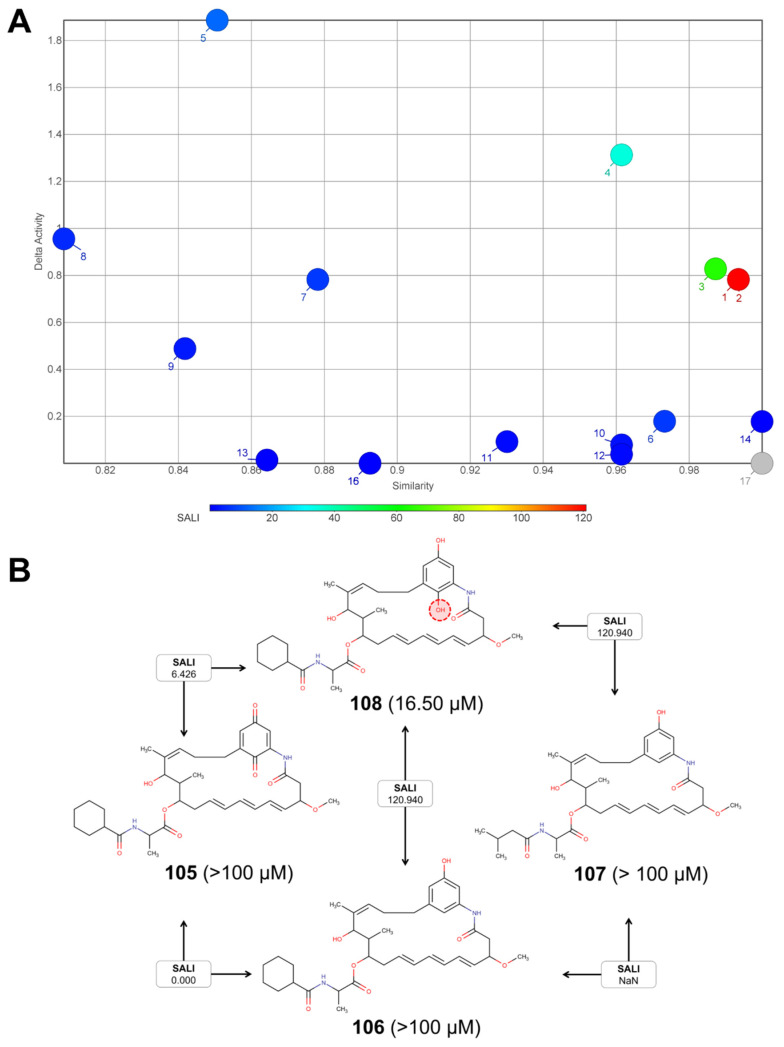
Pairs of structurally related compounds showing activity cliffs. A SALI analysis performed by DataWarrior identifies 17 metabolite pairs with activity cliffs. (**A**) For each pair of compounds, the delta of activity was plotted versus the similarity. The color scale bar indicates the SALI value from 0 (blue) to 120 (red). Each numbered label shows the pair of structurally related compounds listed in Table 5. (**B**) Structures and SALI values for selected compounds (i.e., **105**, **106**, **107** and **108** comprising the pair numbers 1, 2, 7, 15, 16 and 17). The values in parentheses indicate the IC_50_ experimentally calculated for each compound. Compound **108** differs from **105**, **106** and **107** in the additional hydroxyl group at the aromatic ring (highlighted by the red dotted circle).

As the SALI value is directly proportional to the change of the antioxidant potency, a higher SALI value indicated a higher change in bioactivity between very closely related compounds. Likewise, the SALI value coincides with the similarity value between the pair of metabolites. For this reason, 14 to 16 pairs (Table 5) exhibited null SALI values, because the metabolites either had a high similarity or a low potency change. SALI value was not possible to be calculated for the last pair (Table 5, No. 17), since no potency differences were observed for those very structurally related compounds. To visualize these results in a more user-friendly way, a dot-plot that relates the capacity folding (delta activity) against similarity is presented in Figure 11A. In the graph, moving up in each axis indicates more prominent activity cliffs. Hence, pairs 1 to 4 were plotted in the upper-right region. The cluster of structurally related compounds with the highest activity cliffs was taken as a reference to provide details of this measure. In Figure 11B, structures and SALI values for compounds **105**, **106**, **107** and **108** (i.e., pair numbers 1, 2, 7, 15, 16 and 17) are exhibited. The position and orientation of the *para*-hydroxyl groups at the aromatic ring of **108** appear to be an essential feature for the antioxidant capacity. In fact, the additional hydroxyl group appears to activate the other hydroxyl group in the *para*-position to be transferred for antioxidant action, due to the inductive electronic effect. This finding is not surprising because it is recognized that hydrogen-atom transfer is a major mechanism that contributed to the antioxidant capacity of phenolic-like compounds [216]. Additionally, an aromatic mesomeric effect would influence the good antioxidant capacity of **108**, as the *para*-hydroxyl (in *p*-hydroquinone moiety) group easily promotes the formation of a *p*-benzoquinone moiety within an oxidant environment due to its reducing ability. Hence, we explored how the *H*-donor property correlated with the radical scavenging capacity in the whole subgroup of compounds. Likewise, cLogP, cLogS, *H*-acceptors, TPSA, druglikeness and irritant risk properties were included in the examination.

**Figure 12:**
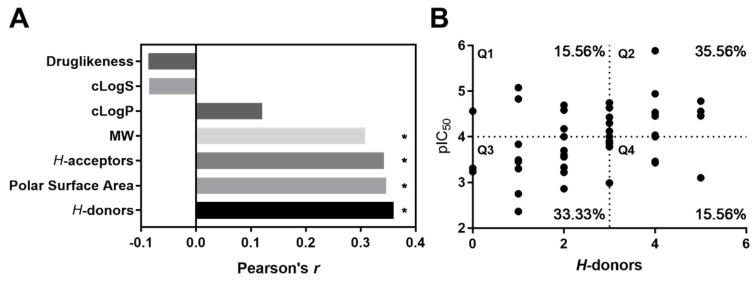
Correlation of molecular properties with antioxidant capacity in *Streptomyces*-derived metabolites. The properties druglikeness, cLogS, cLogP, molecular weight, *H*-acceptors, TPSA and *H*-donor were calculated by DataWarrior. (**A**) Pearson correlation coefficient between antioxidant capacity (pIC_50_) and each calculated property. Asterisk indicates statistically significant correlation. (**B**) Dot plot of antioxidant capacity (pIC_50_) against *H*-donor numbers. To evaluate the association of the property with the activity, a Fisher’s exact test was calculated (*p* = 0.0174). Quadrant 1 (Q1), quadrant 2 (Q2), quadrant 3 (Q3) and quadrant 4 (Q4). In each quadrant, the percent of dots is presented.

Finally, we observed that the molecular descriptors such as molar mass, *H*-acceptors, TPSA and *H*-donors had a significant correlation (*p* < 0.05) with the antioxidant capacity (Figure 12A). It is also important to point out that the *H*-donor property showed a higher correlation with the antioxidant capacity (Figure 12A) and as expected, it explained the function of most compounds (68.9%, *p* = 0,0174) (Figure 12B). Nevertheless, the antioxidant capacity of other compounds (31.1%; Q1 and Q4) was mediated though other mechanisms, given that the exhibited antioxidant effect was regardless of the number of *H*-donors. This disparity may be related to steric accessibility, solubility and electron donors, among other chemical features of the antioxidant agent [217].

## 3. Discussion

Actinobacteria belonging to the genus *Streptomyces* are an important source of bioactive compounds, especially antibiotic agents [39,218]. Furthermore, other industrially relevant bioactivities have been reported for *Streptomyces*-derived metabolites [39,219]. These microorganisms are thus a promising source for new bioactive natural products. Herein, we conducted a systematic literature review to show that *Streptomyces* offers a remarkable opportunity to provide metabolites with photoprotective capabilities, since there is an urgent need to find safer and eco-friendly active ingredients for sunscreens.

We first wanted to illustrate that *Streptomyces* can produce metabolites with photoprotection-related activities. In this literature survey, both crude extracts and pure compounds obtained from several *Streptomyces* strains (89 and 75 strains, respectively) exhibited UV-absorbing, antioxidant, anti-inflammatory properties. However, bearing in mind that few countries have provided studies in this field (Figure 2B), the number of photoprotective metabolite-producing *Streptomyces* strains might be undervalued. Moreover, because *Streptomyces* are ubiquitous microbes found in both terrestrial and marine environments [218,219] and studies involving strains from marine habitats were scarce (34.9%, Figure 3C), it is possible to assume that the real number could be significantly higher. This finding is crucial considering that the biological diversity of *Streptomyces* is widely recognized not only in terms of species numbers but also in terms of the array of secondary metabolite biosynthetic genes [218,220]. Even within a single strain, this diversity may persist [221]. Consequently, investment in research efforts focusing on *Streptomyces* isolates from poorly explored environments thus far (e.g., marine habitats) is highly recommended.

On the other hand, it was found that most studies (65.1%) employed free-living strains, overlooking that the interaction between symbiotic microbes and their hosts is a critical aspect of evolution [222]. Furthermore, these interactions play an influential factor in the evolution of the gene pool for *Streptomyces* specialized metabolism [49]. It is imperative to expand this type of research, involving symbiotic microorganisms from marine environments, as it was evidently the less explored condition (only eight articles evaluated *Streptomyces* isolated from marine organisms—two ascidians [187,189], three corals [184,185,186], one jellyfish [190], one sponge [183] and one starfish [188]).

Regarding chemical diversity, most of the metabolites in the compound library derived from the present review did not show shared fingerprints (Figure 6). This abundance of unique fingerprints may give an idea of the diversity of the gene clusters encoding favorably regulated biosynthetic pathways used by *Streptomyces* to produce metabolites with exclusive structural features for exhibiting both antioxidant and anti-inflammatory properties. This detail is reflected in the different kinds of active *Streptomyces*-derived metabolites reported in the literature (Figure 8). Nevertheless, we found that alkaloids were the most representative group of compounds with antioxidant capacity, while amides were preferably reported as anti-inflammatory compounds. These findings are consistent with several experimental records showing the antioxidant value of alkaloids [223,224,225]. In the same way, several studies have reported amides as naturally occurring anti-inflammatory compounds [226,227,228]. Nonetheless, anti-inflammatory amides exhibiting antioxidant activity have also been reported [229,230], which supports the chance to find compounds having dual antioxidant and anti-inflammatory activities. This fact, in addition to the diversified gene arrays involved in the synthesis of specialized metabolites in *Streptomyces* [220,221], makes these microbes a suitable source to find new and efficient photoprotective molecules.

The biological and chemical diversity of *Streptomyces* is convincing so far. Moreover, *Streptomyces*-derived metabolites also have physicochemical properties and potency comparable with reference or standard agents. For instance, comparing anti-inflammatory compounds with UV filters, the most relevant differences were in druglikeness and irritant risk (Figure 9). However, related to druglikeness, most of the *Streptomyces*-derived compounds fall inside the same interval to that of UV filters (Figure 9D). In contrast, the irritant risk was substantially different. Most of the UV filters were considered high risk, while only a small portion (10%) of *Streptomyces*-derived compounds had the same qualification. This is an important feature because the irritant risk of the UV filters currently available in the market is a contemporary concern [212]. Thus, the fact that *Streptomyces*-derived compounds have been rated as none or low risk is encouraging, as it means that this requirement can be satisfactorily met.

Regarding bioactivity potency, anti-inflammatory compounds were not possible to be analyzed under such a criterion. As inflammation is a physiological response that involves several signaling pathways, there are several different methodological approaches to evaluate the anti-inflammatory activity. This feature was reflected in the literature survey, so it was a factor that limited the analysis options since the biological effects measured for each method could not be compared easily between compounds. On the other hand, the fact that several compounds have shown positive results in a variety of methods also shows that the metabolites might have different ways to produce an anti-inflammatory action. Several compounds were active at micromolar concentrations [145,147,148,162,163,164,175,176,178,181,188,198,199]; even more, some metabolites were as active as reference drugs used in their respective assays. Additionally, a very remarkable case is FK-506 (also known as tacrolimus). This compound was first isolated from *S. tsukubaenis* No. 9993, in 1987 by Kino et al. [151] and currently it is used commercially as a topical anti-inflammatory in dermatology [231]. These findings show that, in terms of anti-inflammatory compounds, *Streptomyces* is an option with a high probability of success at a translational level. In this matter, *Streptomyces* species are especially noteworthy, since their biotechnology potential on an industrial scale has already been proven [232]. Approaches such as metabolic engineering [233], metabolic flux analysis [233] and genetic manipulation [233] have been successful in improving large-scale manufacturing of *Streptomyces*-derived metabolites. Likewise, *Streptomyces* biology knowledge concerning the regulation of secondary metabolite biosynthesis is increasingly more comprehensive [234,235,236,237], which encourages bioprospecting studies on *Streptomyces*, with the opportunity that they will be as successful—in terms of industrial microbes—to produce photoprotective metabolites, as they have been employed for antibiotics production.

Regarding antioxidant compounds, due to the common use of the DPPH radical scavenging assay, it was possible to perform a more detailed analysis by both SPR and SAR examinations. The analysis carried out in the present review allowed us to make evident that some of the *Streptomyces*-derived metabolites were as active or even more active, than well-known reference antioxidants, including vitamin C, vitamin E, quercetin and rutin, among others (Table 4, Figure 10). An antioxidant agent works by the transfer of either a hydrogen atom (HAT), single electron (ET) or both [217]. Accordingly, the methods used to assay potential antioxidant compounds are classified as HAT-based, ET-based or mixed-mode [217]. Some authors consider DPPH radical scavenging assay as a way to assess compounds having a mixed-mode mechanism [238]. In this regard, SALI analysis made it possible to understand that HAT was the mechanism that could be involved in most of the compounds (Figure 12), according to previous publications [216]. However, considering that the results come only from the DPPH radical scavenging assay and bearing in mind that the use of more than one method is strongly encouraged [238], it is not possible to determine the mechanism and, consequently, the actual antioxidant power of these compounds. In this sense, it is important to extend the repertoire of methods to evaluate the antioxidant potential of a certain compound; this would robustly demonstrate its qualification on determining its true potential and scope as an antioxidant. Additionally, a set of distinct parameters should be included, such as kinetics, more than one solvent (e.g., hydrophilic, hydrophobic, protic and aprotic) and different types of radicals and sources. Such a profile can help to comprehensively and accurately determine the antioxidant power of a given compound, in terms of both activity and capacity [238].

The present review is valuable to support the fact that *Streptomyces* are active producers of photoprotective compounds. Nevertheless, their potential as UV-absorbing agents has been poorly studied (2.1% of the publications included in the review). This fact is a meaningful regret, as many of the compounds that were included in this systematic review have conjugated systems, implying a high molar absorption coefficient, which is characteristic of UV filters used for the manufacture of sunscreens [239]. Photoprotective activity, however, goes beyond the UV-absorbing capacity and it is also related to antioxidant and anti-inflammatory activity, which are valuable for effective sunscreen formulations [240,241,242,243]. Mainly, in this study, we provide some evidence insight into the antioxidant capacity of reported compound **108**, which is related to the presence of an active-reducing *para*-hydroquinone-like moiety, implying an interesting starting point for the investigation of the application of *Streptomyces*-derived metabolites as antioxidants. Accordingly, as shown in the present review, it is possible to settle that *Streptomyces* is a bacterial genus with outstanding potential in the development of photoprotective products. Nevertheless, further research needs to be carried out to understand the biology and metabolite production of isolates from different environments and expand the knowledge on the bio and chemodiversity of *Streptomyces*-related microorganisms.

## 4. Methods

### 4.1. Databases and Search Strategy

To achieve as comprehensive a literature examination as possible, the search was conducted using the following databases—Scopus, Web of Science, PubMed, EBSCOhost Research, Scielo and Sage Journals. The terms (including synonyms and related words) and Boolean operators used for each searching were defined as follows:

● Web of Science, PubMed, EBSCOhost Research, Scielo and Sage Journals:

streptomyces AND (“anti UV” OR photoaging OR photoprotection OR “photo protection” OR “sun protection” OR “sunlight protection” OR sunscreen OR sunscreening OR “UV protection” OR “ultraviolet blocking” OR “ultraviolet shielding” OR “UV absorbing”) NOT cyanobacteria

streptomyces AND (antioxidant OR anti-inflammatory)

● Scopus:

streptomyces AND “anti UV” OR photoaging OR photoprotection OR “photo protection” OR “sun protection” OR “sunlight protection” OR sunscreen OR sunscreening OR “UV protection” OR “ultraviolet blocking” OR “ultraviolet shielding” OR “UV absorbing”

streptomyces AND antioxidant OR anti-inflammatory

### 4.2. Selection Procedure

The selection of the articles was based on the following inclusion criteria—(a) original research articles, (b) studies on extracts or compounds derived from identified *Streptomyces* strains and (c) studies evaluating at least one of these activities—UV-absorbing, antioxidant and anti-inflammatory. The following were considered exclusion criteria—(a) articles were written in a language other than English, (b) compounds were not identified and (c) concentrations of compounds or extracts concentrations were not reported.

The article selection process was subdivided into two stages as follows—in the first stage, three researchers separately assessed each title and abstract in a blind process. At this time, an article was marked as potentially eligible to be included in the review when at least two studies indicated that it met the inclusion/exclusion criteria. When an article was indicated by only one researcher, a discussion within the research team was carried out to solve discrepancies. Potentially eligible articles were then examined at the full-text level. At this point, those articles that met the inclusion/exclusion criteria were finally selected for data extraction.

### 4.3. Data Collection and Tabulation

To ensure careful data collection and avoid the risk of bias, before data extraction, a pilot data acquisition form was prepared. The form was evaluated and improved by doing an exercise involving ten randomly selected items. Once the final version of the form was defined, it was used for the data acquisition of the whole set of selected articles. The data were tabulated using the form by one researcher and verified by a second researcher.

### 4.4. Structure-Based Clustering

Compounds retrieved from this systematic review were converted to SMILES notation (simplified molecular-input line-entry system) using Marvin JS (ChemAxon, Budapest, Hungary) to build a custom-made library. Due to the high chemical diversity initially found, these compounds were then clustered according to substructures using binary fingerprints. Hence, the resulting chemical space was initially filtered based on their carbon skeletons and chemical functionalities using the fragment dictionary based binary fingerprint (*FragFp*), path fingerprint (*PathFp)* and organic functionality (*OrgFunctions)* descriptors implemented in Osiris DataWarrior v5.2.1 (Idorsia Pharmaceuticals Ltd., Switzerland) [203]. This substructure filtering led to the exploration of the structural similarity within the custom-made compound library. The detailed inspection of the similarity plots, in combination with metabolic pathway annotation, served as a classification of selected metabolites.

### 4.5. Data Analysis

A meta-analysis was conducted with the metabolites retrieved from the included articles. First, the metabolites were grouped according to their described biological activity. To relate the structural profile and some functional features for a plausible photoprotective-related potential regarding skin penetration and pharmaceutical and sunscreening values, a structure-property relationship analysis (SPR) was first performed. Accordingly, some common physicochemical parameters were then selected, such as molecular weight, topological polar surface area (TPSA), octanol/water partition coefficient (cLogP), aqueous solubility (cLogS), *H*-acceptors and *H*-donors, along with the pharmaceutical criteria druglikeness and the irritant risk was theoretically calculated using the software Osiris DataWarrior v5.2.1. Additionally, the meta-analysis was expanded through a structure-activity relationship analysis (SAR) for those compounds whose antioxidant capacity was assessed by comparable methods to each other. Both analyses were also performed using the software Osiris DataWarrior v5.2.1.

## 5. Conclusions

Bacteria of the genus *Streptomyces* are recognized for their ability to produce a wide variety of bioactive secondary metabolites, most of which show antimicrobial activity. Herein, the *Streptomyces*-derived metabolites with UV-absorbing, anti-inflammatory and antioxidant activities are summarized and their potential as photoprotective compounds is discussed. Although the present survey did not succeed to find any compound possessing all three properties to enhance the photoprotective ability, since this three-component effect was not studied in the original papers for a certain compound, we found that these microbes synthesize compounds with diverse structures, exhibiting a trend related to anti-inflammatory and antioxidant activities. In this sense, good anti-inflammatory results were preferably composed by amides, while the better antioxidant action was produced by alkaloid-type metabolites. On the one hand, regarding anti-inflammatory action, most compounds shared physicochemical properties with FDA-approved UV filters. Unlike these, however, *Streptomyces*-derived metabolites were shown to have a lower risk of being irritants. On the other hand, in the antioxidant compounds, it was seen that the presence of *H*-donors is an essential feature. Despite this, available data do not give a full picture of the actual potential of *Streptomyces* in this field, since there remain significant ecological niches to be explored (e.g., marine symbionts). This work aimed to provide evidence of *Streptomyces* bacteria as promising biofactories of compounds with photoprotective related activities. These compounds may serve as chemical agents to develop safer and eco-friendly sunscreens or other sun protection products.

## Figures and Tables

**Table 1:** Number of *Streptomyces* strains with crude extract screened for photoprotective-related activities.

Source	Bioactivity	No. Strains	Ref.
Terrestrial (Free-living)	Antioxidant	24	[57,58,59,60,61,62,63,64,65,66,67,68,69,70,71,72,73,74,75,76]
	Antioxidant/Anti-inflammatory	2	[77,78,79]
Terrestrial (Symbiont)	Antioxidant	26	[80,81,82,83,84,85,86,87]
	Antioxidant/Anti-inflammatory	1	[88]
Marine (Free-living)	Antioxidant	29	[89,90,91,92,93,94,95,96,97,98,99,100,101,102,103,104,105,106,107,108,109,110,111,112,113]
	Antioxidant/Anti-inflammatory	2	[114,115]
Marine (Symbiont)	Antioxidant	2	[116,117]
NA ^a^	Antioxidant	2	[118]
	Anti-inflammatory	1	[119]
**Total**		**89**	**63**

^a^ Information not available.

**Table 2:** Antioxidant capacity of crude extracts from *Streptomyces* strains.

Strain Name	IC_50_ μg mL^–1^	pIC_50_^a^	Ref.
*Streptomyces* sp. KB3	64.24	4.192	[118]
*Streptomyces* sp. KB1	49.65	4.304	
*Streptomyces* sp. LK3	41.09	4.386	[96]
*Streptomyces* sp. Loyola AR1	750.50	3.125	[62]
*Streptomyces* sp. Loyola UGC	435.31	3.361	[85]
*Streptomyces* sp. MJM 8637	977.20	3.010	[79]
*Streptomyces* sp. MJM 10778	92.80	4.032	[63]
*Streptomyces* sp. OS-6	2.63	5.580	[64]
*Streptomyces* sp. TES-25	46.61	4.332	
*Streptomyces* sp. PS4	950.00	3.022	[65]
*Streptomyces* sp. SC 156	211.20	3.675	[117]
*Streptomyces* sp. SMS_7	609.00	3.215	[103]
*Streptomyces* sp. SMS_SU13	356.00	3.449	
*Streptomyces* sp. SMS_SU21	242.00	3.616	
*Streptomyces* sp. UTMC 1334	45.67	4.340	[104]
*Streptomyces* sp. VITMSS05	92.49	4.034	[67]
*Streptomyces* sp. IB 2014 I 73-1	50.67	4.295	[84]
*Streptomyces* sp. IB 2014 I 73-1HS	33.79	4.471	
*Streptomyces* sp. IB 2014 I 73-2HS	63.39	4.198	
*Streptomyces* sp. IB 2014 I 74-1	57.65	4.239	
*Streptomyces* sp. IB 2014 I 74-2HS	44.22	4.354	
*Streptomyces* sp. IB 2014 I 74-3	32.87	4.483	
*Streptomyces* sp. IB 2014 I 74-4 HS	292.11	3.534	
*Streptomyces* sp. IB 2014 I 74-7HS	6.89	5.162	
*Streptomyces* sp. IB 2014 I 75-1HS	136.86	3.864	
*Streptomyces* sp. IB 2014 I 75-2HS	36.12	4.442	
*Streptomyces* sp. IB 2014 I 75-4HS	1940.70	2.712	
*Streptomyces* sp. IB 2014 I 77-1	310.05	3.509	
*Streptomyces* sp. A071	466.17	3.331	[81]
*S. globosus* A012	97.60	4.011	
*S. hypolithicus* A100	1080.00	2.967	
*S. phaeochromogenes* A009	1325.00	2.878	
*S. carpaticus* MK-01	84.50	4.073	[101]
*S. cellulosae* TES17	2480.00	2.606	[69]
*S. coelicoflavus* BC 01	2.61 ^b^	5.583	[107]
*S. coelicoflavus* BC 02	4.02 ^b^	5.396	
*S. coelicoflavus* BC 04	17.28 ^b^	4.762	
*S. flavoviridis* A3WK	3430.00	2.465	[88]
*S. griesoruber* S2A	860.00	3.066	[108]
*S. hydrogenans* NAF-1	5.58	5.253	[82]
*S. lavendulae* SCA5	507.61	3.294	[74]
*S. nogalater* NIIST A30	30.00	4.523	[76]
*S. omiyaensis* SCH2	2078.13	2.682	[113]
*S. variabilis* DV-35	60.00	4.222	[94]

^a^ Calculated as −logIC_50_(g mL^–1^). ^b^ Calculated from the data shown in the article.

**Table 3:** Number of *Streptomyces* strains and pure compounds screened for photoprotective-related activities.

Source	Bioactivity	No. Strains	No. Compounds	Ref.
Terrestrial (Free-living)	Antioxidant	19	35	[121,122,123,124,125,126,127,128,129,130,131,132,133,134,135,136,137,138,139,140,141]
	Antioxidant/UV-absorbing	2	2	[142,143]
	Anti-inflammatory	8	9	[144,145,146,147,148,149,150,151,152,153,154]
Terrestrial (Symbiotic)	Antioxidant	7	10	[155,156,157,158,159,160,161]
	Anti-inflammatory	5	11	[162,163,164,165,166,167,168]
Marine (Free-living)	Antioxidant	2	2	[169,170]
	Antioxidant/UV-absorbing	1	1	[171]
	Antioxidant/Anti-inflammatory	2	4	[172,173]
	Anti-inflammatory	9	24	[174,175,176,177,178,179,180,181,182]
Marine (Symbiotic)	Antioxidant	3	3	[183,184,185]
	Anti-inflammatory	5	11	[186,187,188,189,190]
NA ^a^	Antioxidant	8	14	[191,192,193,194,195,196,197]
	Anti-inflammatory	4	7	[198,199,200,201,202]
**Total**		**75**	**133**	**82**

^a^ Data not available.

**Table 4:** Antioxidant capacity of *Streptomyces*-derived metabolites.

Compound Number	Molecular Weight (g mol^−1^)	IC_50_ (μM)	pIC_50_^a^	Ref.
**61**	411.342	486.21	3.3132	[202]
**62**	343.223	582.71	3.2345	[202]
**63**	332.446	601.60	3.2207	[202]
**64**	295.421	67.06 ^b^	4.1736	[173]
**65**	411.581	29.33 ^b^	4.5327	[173]
**66**	392.578	27.41 ^b^	4.5621	[173]
**68**	282.422	1770.40	2.7519	[171]
**69**	323.439	25.96 ^b^	4.5858	[143]
**100**	322.315	465.38	3.3322	[214]
**101**	182.174	1372.31	2.8625	[161]
**102**	243.265	1027.69	2.9881	[161]
**103**	574.668	100.00	4.0000	[138]
**104**	576.684	1.30	5.8861	[138]
**105**	636.783	100.00	4.0000	[139]
**106**	622.800	100.00	4.0000	[139]
**107**	596.762	100.00	4.0000	[139]
**108**	638.799	16.50	4.7825	[139]
**113**	324.379	250.00	3.6021	[123]
**114**	340.378	37.20	4.4295	[123]
**115**	486.519	27.70	4.5575	[123]
**117**	150.176	146.49	3.8342	[170]
**118**	346.421	4329.99	2.3635	[124]
**119**	444.391	165.00	3.7825	[125]
**120**	280.381	8.40	5.0757	[126]
**121**	354.576	14.90	4.8268	[126]
**122**	260.337	34.70	4.4597	[126]
**123**	444.526	11.40	4.9431	[127]
**124**	483.607	35.10	4.4547	[127]
**125**	360.361	370.00	3.4318	[128]
**126**	374.388	18.00	4.7447	[128]
**127**	284.270	125.00	3.9031	[197]
**128**	378.335	792.95	3.1008	[185]
**129**	244.245	351.45	3.4541	[157]
**130**	274.271	321.80	3.4924	[157]
**131**	622.924	139.28	3.8561	[158]
**132**	638.923	92.22	4.0352	[158]
**133**	298.293	600.00	3.2218	[159]
**134**	198.177	346.16	3.4607	[160]
**135**	226.183	20.38	4.6908	[161]
**136**	206.196	277.02	3.5575	[161]
**137**	138.122	200.00	3.6990	[129]
**138**	168.191	500.00	3.3010	[131]
**139**	212.204	50.30	4.2984	[132]
**140**	198.177	75.80	4.1203	[132]
**141**	207.184	23.26	4.6334	[134]

^a^ Calculated as −logIC_50_(M). ^b^ Calculated from the data shown in the article.

**Table 5:** Structure-Activity Landscape Index (SALI) analysis of structurally related compounds with antioxidant capacity.

Pair No	ID 1	ID 2	Similarity	Activity 1	Activity 2	Delta Activity	SALI
1	**106**	**108**	0.994	4.000	4.783	0.783	120.940
2	**107**	**108**	0.994	4.000	4.783	0.783	120.940
3	**113**	**114**	0.987	3.602	4.429	0.827	64.979
4	**125**	**126**	0.962	3.432	4.745	1.313	34.102
5	**103**	**104**	0.851	4.000	5.886	1.886	12.630
6	**131**	**132**	0.973	3.856	4.035	0.179	6.710
7	**105**	**108**	0.878	4.000	4.783	0.783	6.426
8	**113**	**115**	0.809	3.602	4.558	0.955	4.993
9	**123**	**124**	0.842	4.943	4.455	0.488	3.087
10	**61**	**62**	0.962	3.313	3.235	0.079	2.042
11	**61**	**63**	0.930	3.313	3.221	0.092	1.323
12	**129**	**130**	0.962	3.454	3.492	0.038	0.994
13	**62**	**63**	0.864	3.235	3.221	0.014	0.102
14	**139**	**140**	1.000	4.298	4.120	0.178	0.000
15	**105**	**107**	0.893	4.000	4.000	0.000	0.000
16	**105**	**106**	0.893	4.000	4.000	0.000	0.000
17	**106**	**107**	1.000	4.000	4.000	0.000	NaN ^a^

^a^ NaN, Not a Number.

## Supplementary Materials

The following are available online at https://www.mdpi.com/1420-3049/25/14/3221/s1, Table S1: PRISMA checklist, Table S2: List of compounds retrieved from the included papers, Table S3: *Streptomyces* strains source of bioactive crude extracts, Table S4: Chemical structures of retrieved compounds, Table S5: Databases search results, Figure S1: The number of papers in the literature by country of *Streptomyces* strain origin.
