# Supplementary material for: Streptomyces-Derived Metabolites with Potential Photoprotective Properties—A Systematic Literature Review and Meta-Analysis on the Reported Chemodiversity
Source: Molecules. 2020 Jul 15;25(14):3221. doi: 10.3390/molecules25143221 (PMC7397340; doi:10.3390/molecules25143221)
Supplement: Supplementary file 1 [file molecules-25-03221-s001.zip › Supplementary Materials/Figure_S1.docx]

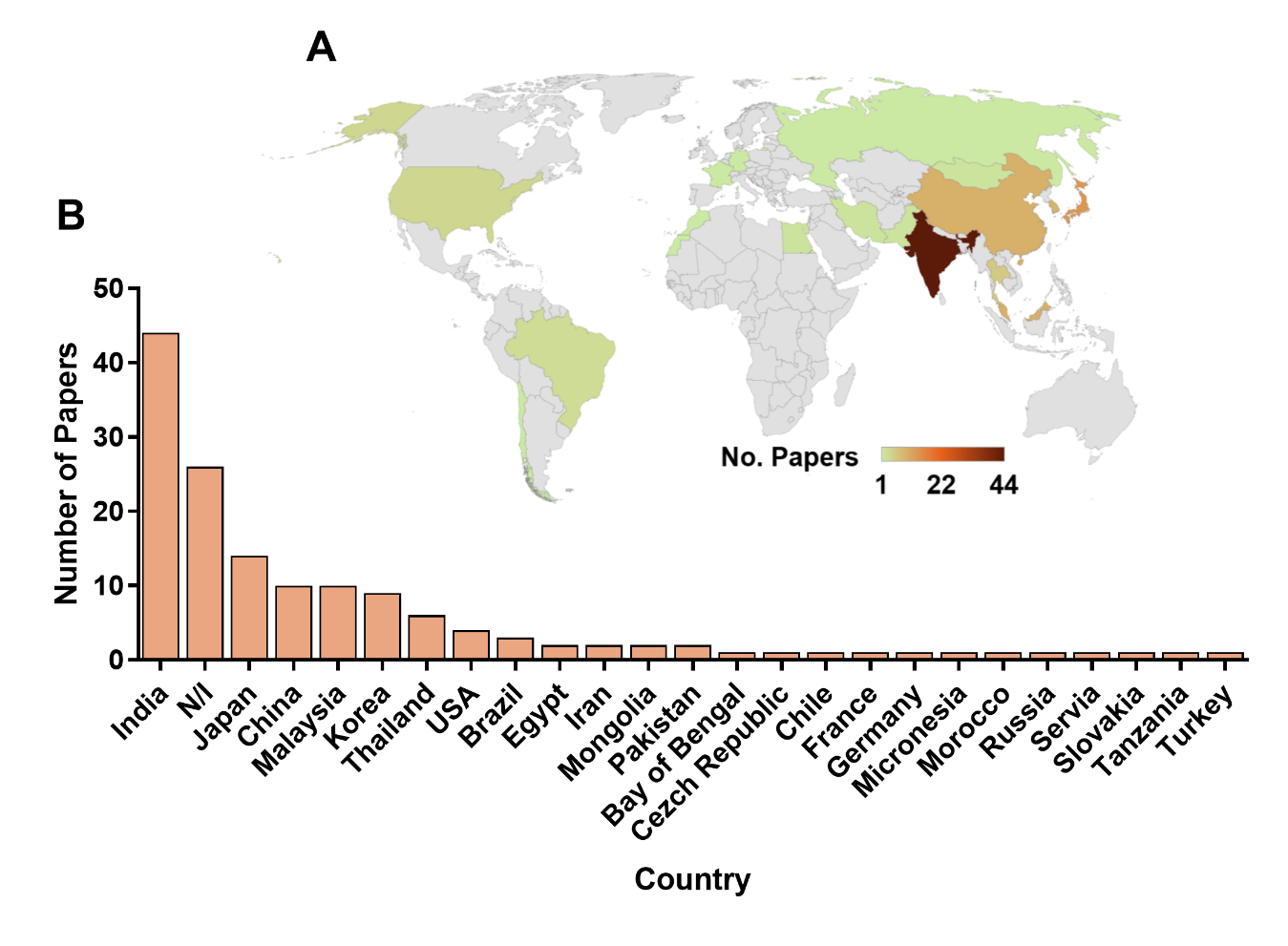


**Figure S1. The number of papers in the literature by country of *Streptomyces* strain origin.** (A) World map showing the countries where the *Streptomyces* strains were sampled. (B) Publication frequency by the country where the Streptomyces strains were sampled. N/I: Not informed.
