# Supplementary material for: Streptomyces-Derived Metabolites with Potential Photoprotective Properties—A Systematic Literature Review and Meta-Analysis on the Reported Chemodiversity
Source: Molecules. 2020 Jul 15;25(14):3221. doi: 10.3390/molecules25143221 (PMC7397340; doi:10.3390/molecules25143221)
Supplement: Supplementary file 1 [file molecules-25-03221-s001.zip › Supplementary Materials/Table_S2.docx]

**Table S2.** List of compounds retrieved from the included papers.

| **Comp No.** | **Compound Name** | **SMILES^a^** | ***Streptomyces* strain** | **Bioactivity tested** | **Source type** | **Reference** |
| --- | --- | --- | --- | --- | --- | --- |
| **1** | Axinelline A | CCOC(=O)[C@H](CO)NC(=O)C1=CC=CC(O)=C1O | *S. axinellae* | Anti-inflammatory | Marine (Symbiont) | [1] |
| **2** | Lawsonone | O[C@@H]1COCC(C1)C(=O)COC1=CC(=O)C2=CC=CC=C2C1=O | *Streptomyces* sp. Lawsonone | Anti-inflammatory | Marine (Symbiont) | [2] |
| **3** | Succinilenes A | CC[C@@H](O)[C@H](O)C\C=C\C=C\C=C\C[C@H](O)C(\C)=C\[C@@H](C)[C@@H](C)OC(=O)CCC(O)=O | *S. flavogriseus* | Anti-inflammatory | Marine (Free-living) | [3] |
| **4** | Succinilenes B | CC[C@H](O)[C@H](O)C\C=C\C=C\C=C\C[C@H](O)C(\C)=C\[C@@H](C)[C@@H](C)OC(=O)CCC(O)=O | *S. flavogriseus* | Anti-inflammatory | Marine (Free-living) | [3] |
| **5** | Succinilenes C | CCC(=O)[C@H](O)C\C=C\C=C\C=C\C[C@H](O)C(\C)=C\[C@@H](C)[C@@H](C)OC(=O)CCC(O)=O | *S. flavogriseus* | Anti-inflammatory | Marine (Free-living) | [3] |
| **6** | S632A3 | [H][C@@](O)(CC1CC(=O)NC(=O)C1)CC(=O)[C@@]([H])(C)\C=C(/C)\C=C\C | *S. hygroscopicus* S632 | Anti-inflammatory | Terrestrial (Free-living) | [4] |
| **7** | 9-methylstreptimidone | C\C=C/C(/C)=C/[C@@H](C)C(=O)C[C@H](O)CC1CC(=O)NC(=O)C1 | N/I | Anti-inflammatory | N/I | [5] |
| **8** | YC-17 | CC[C@H]1OC(=O)[C@H](C)[C@@H](O[C@@H]2O[C@H](C)C[C@@H]([C@H]2O)N(C)C)[C@@H](C)C[C@@H](C)C(=O)\C=C\[C@H]1C | *S. venezuelae* ATCC 15439 | Anti-inflammatory | N/I | [6] |
| **9** | Actinoquinoline A | CC(C)CC(=O)NC[C@H](O)CC[C@@H](CO)NC(=O)C1=NC2=CC=CC=C2C=C1O | *Streptomyces* sp. CNP975 | Anti-inflammatory | Marine (Free-living) | [7] |
| **10** | Actinoquinoline B | CC(C)CC(=O)NC[C@H]1CC[C@H](NC(=O)C2=NC3=CC=CC=C3C=C2O)[C@H](O)O1 | *Streptomyces* sp. CNP975 | Anti-inflammatory | Marine (Free-living) | [7] |
| **11** | Camporidine A | CCCCCCC1CNC2C=C\C(=C/C=C/C(O)=O)C22OC12 | *Streptomyces* sp. STA1. | Anti-inflammatory | Terrestrial (Symbiont) | [8] |
| **12** | Anmindenol A | CC(C)\C=C1/C=C(CO)C2=CC=C(C)C=C12 | *Streptomyces* sp. CMDD10D111 | Anti-inflammatory | Marine (Free-living) | [9] |
| **13** | Anmindenol B | CC(C)\C=C1/C[C@](O)(CO)C2=CC=C(C)C=C12 | *Streptomyces* sp. CMDD10D111 | Anti-inflammatory | Marine (Free-living) | [9] |
| **14** | Violapyrone B | CC(C)CCCCC1=CC(O)=C(C)C(=O)O1 | *Streptomyces* sp. 112CH148 | Anti-inflammatory | Marine (Symbiont) | [10] |
| **15** | Violapyrone C | CC[C@H](C)CCCCC1=CC(O)=C(C)C(=O)O1 | *Streptomyces* sp. 112CH148 | Anti-inflammatory | Marine (Symbiont) | [10] |
| **16** | Griseusrazin A | [H]C(=O)N(C)C1=CC=C(CC2=NC(C)=C(CC3=CC=C(C=C3)N(C)C([H])=O)N=C2C)C=C1 | *S. griseus* 09-0144 | Anti-inflammatory | Marine (Free-living) | [11] |
| **17** | Somalimycin (1) | CC(C)C(=O)O[C@H]1[C@H](C)OC(=O)[C@@H](NC(=O)C2=CC=CC(N)=C2O)[C@@H](C)OC(=O)[C@@H]1C | *S. somaliensis* SCSIO ZH66 | Anti-inflammatory | Marine (Free-living) | [12] |
| **18** | USF-19A (2) | [H]C(=O)NC1=C(O)C(=CC=C1)C(=O)N[C@H]1[C@@H](C)OC(=O)[C@H](C)[C@@H](OC(=O)C(C)C)[C@H](C)OC1=O | *S. somaliensis* SCSIO ZH66 | Anti-inflammatory | Marine (Free-living) | [12] |
| **19** | Urauchimycin D (3) | [H]C(=O)NC1=C(O)C(=CC=C1)C(=O)N[C@H]1[C@@H](C)OC(=O)[C@H](C)[C@@H](O)[C@H](C)OC1=O | *S. somaliensis* SCSIO ZH66 | Anti-inflammatory | Marine (Free-living) | [12] |
| **20** | UK-2A | COC1=C(O)C(=NC=C1)C(=O)N[C@H]1COC(=O)[C@H](CC2=CC=CC=C2)[C@@H](OC(=O)C(C)C)[C@H](C)OC1=O | *Streptomyces* sp. 517-02 | Anti-inflammatory | Terrestrial (Free-living) | [13] |
| **21** | Splenocin A | [H]C(=O)NC1=C(O)C(=CC=C1)C(=O)N[C@H]1[C@@H](C)OC(=O)[C@H](CC2=CC=CC=C2)[C@@H](OC(=O)CC)[C@H](C)OC1=O | *Streptomyces* sp. CNQ431 | Anti-inflammatory | Marine (Free-living) | [14] |
| **22** | Splenocin B | [H]C(=O)NC1=C(O)C(=CC=C1)C(=O)N[C@H]1[C@@H](C)OC(=O)[C@H](CC2=CC=CC=C2)[C@@H](OC(=O)C(C)C)[C@H](C)OC1=O | *Streptomyces* sp. CNQ431 | Anti-inflammatory | Marine (Free-living) | [14] |
| **23** | Splenocin C | [H]C(=O)NC1=C(O)C(=CC=C1)C(=O)N[C@H]1[C@@H](C)OC(=O)[C@H](CC2=CC=CC=C2)[C@@H](OC(=O)C(C)CC)[C@H](C)OC1=O | *Streptomyces* sp. CNQ431 | Anti-inflammatory | Marine (Free-living) | [14] |
| **24** | Splenocin D | [H]C(=O)NC1=C(O)C(=CC=C1)C(=O)N[C@H]1[C@@H](C)OC(=O)[C@H](CC)[C@@H](OC(=O)C2=CC=CC=C2)[C@H](C)OC1=O | *Streptomyces* sp. CNQ431 | Anti-inflammatory | Marine (Free-living) | [14] |
| **25** | Splenocin E | [H]C(=O)NC1=C(O)C(=CC=C1)C(=O)N[C@H]1[C@@H](C)OC(=O)[C@H](CCCC)[C@@H](OC(=O)C2=CC=CC=C2)[C@H](C)OC1=O | *Streptomyces* sp. CNQ431 | Anti-inflammatory | Marine (Free-living) | [14] |
| **26** | Splenocin F | [H]C(=O)NC1=C(O)C(=CC=C1)C(=O)N[C@H]1[C@@H](C)OC(=O)[C@H](CCCCC)[C@@H](OC(=O)C2=CC=CC=C2)[C@H](C)OC1=O | *Streptomyces* sp. CNQ431 | Anti-inflammatory | Marine (Free-living) | [14] |
| **27** | Splenocin G | [H]C(=O)NC1=C(O)C(=CC=C1)C(=O)N[C@H]1[C@@H](C)OC(=O)[C@H](CCCCCC)[C@@H](OC(=O)C2=CC=CC=C2)[C@H](C)OC1=O | *Streptomyces* sp. CNQ431 | Anti-inflammatory | Marine (Free-living) | [14] |
| **28** | Splenocin H | [H]C(=O)NC1=C(O)C(=CC=C1)C(=O)N[C@H]1[C@@H](C)OC(=O)[C@H](CC(C)CCCC)[C@@H](OC(=O)C2=CC=CC=C2)[C@H](C)OC1=O | *Streptomyces* sp. CNQ431 | Anti-inflammatory | Marine (Free-living) | [14] |
| **29** | Splenocin I | [H]C(=O)NC1=C(O)C(=CC=C1)C(=O)N[C@H]1[C@@H](C)OC(=O)[C@H](CC2=CC=CC=C2)[C@@H](OC(=O)C2=CC=CC=C2)[C@H](C)OC1=O | *Streptomyces* sp. CNQ431 | Anti-inflammatory | Marine (Free-living) | [14] |
| **30** | Splenocin J | [H]C(=O)NC1=C(O)C(=CC=C1)C(=O)N[C@H]1[C@@H](C)OC(=O)[C@H](CC2=CC=CC=C2)[C@@H](O)[C@H](C)OC1=O | *Streptomyces* sp. CNQ431 | Anti-inflammatory | Marine (Free-living) | [14] |
| **31** | (R)-7-acetyl-3,6-dihydroxy-8-propyl-3,4-dihydronaphthalen-1(2H)-one | CCCC1=C2C(=O)C[C@H](O)CC2=CC(O)=C1C(C)=O | *S. violaceoruber* YIM 101131 | Anti-inflammatory | Terrestrial (Symbiont) | [15] |
| **32** | Actinofuranone G | CC(O)\C=C(/C)[C@H](O)[C@H](C)\C=C\C=C\CC[C@@H](O)CC1=C(C)C(=O)C(O)(O1)C(C)O | *S. gramineus* YIM 130461 | Anti-inflammatory | Terrestrial (Symbiont) | [16] |
| **33** | Actinofuranone H | CC\C=C(/C)[C@H](O)[C@H](C)\C=C\C=C\C(O)C[C@@H](O)CC1=C(C)C(=O)C(O)(O1)C(C)O | *S. gramineus* YIM 130461 | Anti-inflammatory | Terrestrial (Symbiont) | [16] |
| **34** | E-975 | CC\C=C(/C)[C@H](O)[C@H](C)\C=C\C=C\CC[C@@H](O)CC1=C(C)C(=O)C(O)(O1)C(C)O | *S. gramineus* YIM 130461 | Anti-inflammatory | Terrestrial (Symbiont) | [16] |
| **35** | E-492 | C\C=C(/C)[C@H](O)[C@H](C)\C=C\C=C\CC[C@@H](O)CC1=C(C)C(=O)C(O)(O1)C(C)O | *S. gramineus* YIM 130461 | Anti-inflammatory | Terrestrial (Symbiont) | [16] |
| **36** | Violacin A | CC(=O)CC1(O)CC(=O)C2=C(C)C=C(O)C=C2O1 | *S. violaceoruber* YIM 101131 | Anti-inflammatory | Terrestrial (Symbiont) | [17] |
| **37** | Cyclo(L-Leu-Trans-4-Hydroxy-L-Pro) | [H][C@@]12C[C@@H](O)CN1C(=O)[C@H](CC(C)C)NC2=O | *Streptomyces* sp. NIO-1034 | Anti-inflammatory | Marine (Symbiont) | [18] |
| **38** | ZINC13416156 | [H][C@]12CCCN1C(=O)[C@@]([H])(NC2=O)[C@H](C)CC | *Streptomyces* sp. NIO-1034 | Anti-inflammatory | Marine (Symbiont) | [18] |
| **39** | Cyclo(L-Phe-Trans-4-Hydroxy-L-Pro) | [H][C@@]12C[C@@H](O)CN1C(=O)[C@H](CC1=CC=CC=C1)NC2=O | *Streptomyces* sp. NIO-1034 | Anti-inflammatory | Marine (Symbiont) | [18] |
| **40** | Cyclo(-D-Leu-D-Pro) | [H][C@]12CCCN1C(=O)[C@@H](CC(C)C)NC2=O | *Streptomyces* sp. NIO-1034 | Anti-inflammatory | Marine (Symbiont) | [18] |
| **41** | Cyclo(D-Pro-L-Phe-) | [H][C@]12CCCN1C(=O)[C@H](CC1=CC=CC=C1)NC2=O | *Streptomyces* sp. NIO-1034 | Anti-inflammatory | Marine (Symbiont) | [18] |
| **42** | Colabomycin E | C\C=C\C=C/C=C/C=C/C=C/C(=O)CNC1=CC(O)(\C=C\C=C\C=C\C=C\C(=O)NC2C(O)CCC2=O)C2OC2C1=O | *S. aureus* SOK1/5-04 | Anti-inflammatory | Terrestrial (Free-living) | [19] |
| **43** | Strepsesquitriol | C[C@H]1C[C@@H](O)[C@]23CC(CC[C@]12O)C(C)(C)[C@]3(C)O | *Streptomyces* sp. SCSIO 10355 | Anti-inflammatory | Marine (Free-living) | [20] |
| **44** | Phaeochromycin A | CCCC1=CC(O)=C2C(O)=CC=CC2=C1C1=CC(O)=CC(=O)O1 | *S. phaeochromogenes* LL-P018 | Anti-inflammatory | Terrestrial (Free-living) | [21] |
| **45** | Phaeochromycin C | CCCC1=CC(=O)C2=C(CC3=CC(O)=CC(=O)O3)C=CC=C2O1 | *S. phaeochromogenes* LL-P018 | Anti-inflammatory | Terrestrial (Free-living) | [21] |
| **46** | SCH 36605 | CC(C)CC(N)C(=O)NC(CCN(C)C(N)=N)CC(=O)NC1C=CC(OC1C(O)=O)N1C=C(CO)C(N)=NC1=O | *Streptomyces* sp. SCH 36605 | Anti-inflammatory | Terrestrial (Free-living) | [22] |
| **47** | SEK-1005 | CC(C)CC1CCC(O)(OC1C)C(C)(O)C(=O)NC1C(OC(=O)C(CO)NC(=O)C2CCCNN2C(=O)C(CC2=CC=CC=C2)N(C)C(=O)C(C)N(O)C(=O)C2CCCNN2C1=O)C(C)C | *S. nobilis* | Anti-inflammatory | N/I | [23] |
| **48** | *FK-506* | CO[C@@H]1C[C@@H](CC[C@H]1O)\C=C(/C)[C@H]1OC(=O)[C@@H]2CCCCN2C(=O)C(=O)[C@]2(O)O[C@@H]([C@H](C[C@H]2C)OC)[C@H](C[C@@H](C)C\C(C)=C\[C@@H](CC=C)C(=O)C[C@H](O)[C@H]1C)OC | *S. tsukubaenis* No. 9993 | Anti-inflammatory | Terrestrial (Free-living) | [24–27] |
| **49** | Dianemycin | [H][C@@]1(C[C@@](O)(CO)[C@H](C)C[C@@H]1C)C1C[C@H](C)[C@]2(O1)OC(C[C@H](O[C@H]1CC[C@H](OC)[C@@H](C)C1)[C@H]2C)[C@]1(C)CC[C@]2(C[C@H](O)[C@@H](C)[C@]([H])(O2)[C@@H](C)\C=C(/C)C(=O)[C@H](C)CC(C)C(O)=O)O1 | *Streptomyces* sp. MT 2705-4 | Anti-inflammatory | Terrestrial (Free-living) | [28] |
| **50** | Nonactin | [H][C@@]12CC[C@@]([H])(O1)[C@@H](C)C(=O)O[C@H](C)C[C@@]1([H])CC[C@]([H])(O1)[C@H](C)C(=O)O[C@@H](C)C[C@]1([H])CC[C@@]([H])(O1)[C@@H](C)C(=O)O[C@H](C)C[C@@]1([H])CC[C@]([H])(O1)[C@H](C)C(=O)O[C@@H](C)C2 | *S. griseus* | Anti-inflammatory | N/I | [29] |
| **51** | WS-7528 | CC1C(OC2=C(C(O)=CC(O)=C2)C1=O)C1=CC=C(O)C=C1 | *Streptomyces* sp. 7528 | Anti-inflammatory | Terrestrial (Free-living) | [30] |
| **52** | 5,7-dimethyloxy-4-p-methoxylphenylcoumarin | COC1=CC=C(C=C1)C1=CC(=O)OC2=CC(OC)=CC(OC)=C12 | *S. aureofaciens* CMUAc130 | Anti-inflammatory | Terrestrial (Symbiont) | [31] |
| **53** | 5,7-dimethoxy-4-phenylcoumarin | COC1=CC(OC)=C2C(OC(=O)C=C2C2=CC=CC=C2)=C1 | *S. aureofaciens* CMUAc130 | Anti-inflammatory | Terrestrial (Symbiont) | [31] |
| **54** | Lansai C | CC(C)\C=C1\N(C)C(=O)\C(=C/C2=CC=CC=C2)N(O)C1=O | *Streptomyces* sp. SUC1 | Anti-inflammatory | Terrestrial (Symbiont) | [32] |
| **55** | Lansai D | CC(C)\C=C1\N(C)C(=O)\C(NC1=O)=C/C1=CC=CC=C1 | *Streptomyces* sp. SUC1 | Anti-inflammatory | Terrestrial (Symbiont) | [33] |
| **56** | Thienodolin | NC(=O)C1=CC2=C(NC3=CC(Cl)=CC=C23)S1 | *Streptomyces* sp. CNY-325 | Anti-inflammatory | Marine (Free-living) | [34] |
| **57** | Salinamide A | CC[C@H](C)C1C(=O)N[C@H]2C3=CC=C(C=C3)O[C@@H]([C@@]4(CO4)/C=C\C(=O)NCC(=O)OC[C@@H](C(=O)O[C@@H]([C@@H](C(=O)N1)NC(=O)[C@H](C)[C@@H](C(C)C)O)C)NC(=O)[C@H](NC(=O)[C@@H](N(C2=O)C)CC5=CC=CC=C5)[C@@H](C)O)C | *Streptomyces* sp. CNB091 | Anti-inflammatory | Marine (Symbiont) | [35] |
| **58** | Salinamide B. | CC[C@H](C)[C@@H]1C(=O)N[C@H]2C3=CC=C(C=C3)O[C@@H]([C@](/C=C\C(=O)NCC(=O)OC[C@@H](C(=O)O[C@@H]([C@@H](C(=O)N1)NC(=O)[C@H](C)[C@@H](C(C)C)O)C)NC(=O)[C@H](NC(=O)[C@@H](N(C2=O)C)CC4=CC=CC=C4)[C@@H](C)O)(CCl)O)C | *Streptomyces* sp. CNB091 | Anti-inflammatory | Marine (Symbiont) | [35] |
| **59** | Cyclomarin A | COC([C@@H]1NC(=O)[C@H](C)NC(=O)C(C[C@@H](C)CO)N(C)C(=O)[C@@H](NC(=O)[C@@H](NC(=O)[C@H](CC(C)C)N(C)C(=O)[C@@H](NC1=O)C(C)C)[C@H](C)C=C(C)C)[C@H](O)C1=CN(C2=C1C=CC=C2)C(C)(C)C1CO1)C1=CC=CC=C1 | *Streptomyces* sp. CNB-382 | Anti-inflammatory | Marine (Free-living) | [36] |
| **60** | Caniferolide A | CCC(OC1CC(C)(O)C(OC2CCC(OC(=O)C(C)C(O)C3=CC4=C(C=C3)C(=O)C(C)CC4=O)C(C)O2)C(C)O1)C(C)C(O)C(C)C(O)C(C)C1OC(=O)\C=C\C(C)C(O)CC(O)CCCC(O)CCC(C)C(O)C(=O)C2(O)OC(CC(OS(O)(=O)=O)C2O)CC(O)CC(O)CC(O)CCCC(O)C2OC2C1C | *S. caniferus* | Antioxidant/Anti-inflammatory | Marine (Free-living) | [37] |
| **61** | Phenazine 1 | CC1=C(CN2C3=CC=CC=C3N=C3C(=O)C(Br)=CC=C23)CCC(C)(C)C1 | *Streptomyces* sp. CNS284 | Antioxidant/Anti-inflammatory | N/I | [38] |
| **62** | Phenazine 2 | CC(C)=CCN1C2=CC=CC=C2N=C2C(=O)C(Br)=CC=C12 | *Streptomyces* sp. CNS284 | Antioxidant/Anti-inflammatory | N/I | [38] |
| **63** | Lavanducyanin 3 | CC1=C(CN2C3=CC=CC=C3N=C3C(=O)C=CC=C23)CCC(C)(C)C1 | *Streptomyces* sp. CNS284 | Antioxidant/Anti-inflammatory | N/I | [38] |
| **64** | BC 01_C1 | CCCC(CCO)CCC(=O)CCC1=CCC(N)CC1=O | *S. coelicoflavus* BC 01 | Antioxidant/Anti-inflammatory | Marine (Free-living) | [39] |
| **65** | BC 01_C2 | CC(COCO)C(C)C(O)C1C(C)CCC2C1CC(=O)C1C(CN)C(O)CCC21 | *S. coelicoflavus* BC 01 | Antioxidant/Anti-inflammatory | Marine (Free-living) | [39] |
| **66** | BC 01_C3 | CCCC\C(CC)=C\OC(=O)C1CCCCC1C(=O)OC\C(CCCC)=C\C | *S. coelicoflavus* BC 01 | Antioxidant/Anti-inflammatory | Marine (Free-living) | [39] |
| **67** | Undecylprodigiosin | CCCCCCCCCCCC1=CC=C(N1)\C=C1/N=C(C=C1OC)C1=CC=CN1 | *Streptomyces* sp. JS520 | Antioxidant/UV-absorbing | Terrestrial (Free-living) | [40] |
| **68** | 2R-Acetoxymethyl-1,3,3-trimethyl-4t-(3-methyl-2-buten-1-yl)-1t-cyclohexanol | CC(C)=CCC1CCC(C)(O)C(COC(C)=O)C1(C)C | *Streptomyces* sp. VITJS8 | Antioxidant/UV-absorbing | Marine (Free-living) | [41] |
| **69** | Prodigiosin | CCCCCC1=C\C(=C\C2=C(OC)C=C(N2)C2=CC=CN2)N=C1C | *Streptomyces* sp. WMA-LM31 | Antioxidant/UV-absorbing | Terrestrial (Free-living) | [42] |
| **70** | metacycloprodigiosin | CC[C@@H]1CCCCCCCCC2=CC1=C(N2)\C=C1/N=C(C=C1OC)C1=CC=CN1 | *Streptomyces* sp. CBS 198.65 | Antioxidant | N/I | [43] |
| **71** | Ageloline A | OC(=O)C1=CC(=O)C2=C(N1)C=C(Cl)C=C2 | *Streptomyces* sp. SBT345 | Antioxidant | Marine (Symbiont) | [44] |
| **72** | K-563 | CC(C)CCCCCCCCCCCN(C)OP(O)(=O)N(CC(N)=O)P(O)(=O)OC1C(O)C(O)C(CO)=C1OCC1OC(C(O)C1O)N1C=CC(N)=NC1=O | *Streptomyces* sp. 3728‐17 | Antioxidant | Terrestrial (Symbiont) | [45] |
| **73** | (-)-8-O-methyltetrangomycin | COC1=CC=CC2=C1C(=O)C1=C(C2=O)C2=C(C[C@@](C)(O)CC2=O)C=C1 | *Streptomyces* sp. AC37 | Antioxidant | Terrestrial (Symbiont) | [46] |
| **74** | Salinomycin | [H][C@@]1(CC[C@H](C)[C@@]([H])(O1)[C@@H](C)[C@H](O)[C@H](C)C(=O)[C@H](CC)[C@@]1([H])O[C@@]2(O[C@@]3(CC[C@](C)(O3)[C@@]3([H])CCC(O)(CC)[C@H](C)O3)[C@H](O)C=C2)[C@H](C)C[C@@H]1C)[C@@H](CC)C(O)=O | *S. albus* | Antioxidant | N/I | [47] |
| **75** | 3-epi- 5-deoxyenterocin | [H][C@@]1(C(=O)C2=CC=CC=C2)C2(O)CC3CC(O)(C2C2=CC(OC)=CC(=O)O2)[C@]1(O)C(=O)O3 | *Streptomyces* C1 | Antioxidant | N/I | [43] |
| **76** | 5-deoxyenterocin | COC1=CC(=O)OC(=C1)C1C2(O)CC3OC(=O)[C@]4(O)[C@@H](C(=O)C5=CC=CC=C5)C1(O)CC234 | *Streptomyces* C1 | Antioxidant | N/I | [43] |
| **77** | anhydroexfoliamycin | CCCC1OC(C)CC2=CC3=C(C(O)=C12)C(=O)C(=CC3=O)C1OC(CO)C(O)C1O | *Streptomyces* sp. Lt 005 | Antioxidant | N/I | [43] |
| **78** | naphthopyranomycin | CCCC1OC(C)=CC2=CC3=C(C(O)=C12)C(=O)C([C@@H]1OC[C@@H](O)[C@H](CO)O1)=C(CC(C)=O)C3=O | *Streptomyces* sp. Lt 005 | Antioxidant | N/I | [43] |
| **79** | nocardamine | ON1CCCCCNC(=O)CCC(=O)N(O)CCCCCNC(=O)CCC(=O)N(O)CCCCCNC(=O)CCC1=O | *Streptomyces* C1 | Antioxidant | N/I | [43] |
| **80** | Pyrrolostatin | CC(C)=CCC\C(C)=C\CC1=CNC(=C1)C(O)=O | *S. chrestomyceticus* EC40 | Antioxidant | Terrestrial (Free-living) | [48] |
| **81** | Benzastatin A | COC\C(CCC(C)=C(C)C)=C/CC1=CC(=CC=C1N)C(N)=O | *S. nitrosporeus* 30643 | Antioxidant | Terrestrial (Free-living) | [49] |
| **82** | Benzastatin B | CC(C)=C(C)CC\C(C)=C\CC1=CC(=CC=C1N)C(N)=O | *S. nitrosporeus* 30643 | Antioxidant | Terrestrial (Free-living) | [49] |
| **83** | Benzastatin C | COC[C@@]1(CCC(C)=C(C)C)NC2=CC=C(C=C2C[C@H]1Cl)C(N)=O | *S. nitrosporeus* 30643 | Antioxidant | Terrestrial (Free-living) | [49] |
| **84** | Benzastatin D | COC[C@@]1(CCC(C)=C(C)C)NC2=CC=C(C=C2C[C@H]1O)C(N)=O | *S. nitrosporeus* 30643 | Antioxidant | Terrestrial (Free-living) | [49] |
| **85** | 3',4',7-trihydroxyisoflavone (2) | OC1=CC2=C(C=C1)C(=O)C(=CO2)C1=CC(O)=C(O)C=C1 | *Streptomyces* sp. OH-1049 | Antioxidant | Terrestrial (Free-living) | [50] |
| **86** | 4',7,8-trihydroxyisoflavone (1) | OC1=CC=C(C=C1)C1=COC2=C(C=CC(O)=C2O)C1=O | *Streptomyces* sp. OH-1049 | Antioxidant | Terrestrial (Free-living) | [50] |
| **87** | 8-chloro-3',4',5,7-tetrahydroxyisoflavone (3) | OC1=CC(O)=C(Cl)C2=C1C(=O)C(=CO2)C1=CC(O)=C(O)C=C1 | *Streptomyces* sp. OH-1049 | Antioxidant | Terrestrial (Free-living) | [50] |
| **88** | Stealthin A | NC1=C2C(C3=C1C=C(CO)C=C3O)=C(O)C1=C(C(O)=CC=C1)C2=O | *S. viridochromogenes* | Antioxidant | N/I | [51] |
| **89** | Stealthin B | NC1=C2C(C3=C1C=C(C=O)C=C3O)=C(O)C1=C(C(O)=CC=C1)C2=O | *S. viridochromogenes* | Antioxidant | N/I | [51] |
| **90** | Carquinostatin A | C[C@@H](O)CC1=C(C)C(=O)C(=O)C2=C1NC1=C2C=C(CC=C(C)C)C=C1 | *S. exfoliatus* 2419-SVT2 | Antioxidant | N/I | [52] |
| **91** | Naphterpin B | [H][C@]12CC[C@@](C)(O)C=C1C1=C(OC2(C)C)C(=O)C2=C(C=C(O)C(C)=C2O)C1=O | *Streptomyces* sp. CL190 | Antioxidant | N/I | [53] |
| **92** | Naphterpin C | [H][C@]12CC[C@](C)(O)C=C1C1=C(OC2(C)C)C(=O)C2=C(C=C(O)C(C)=C2O)C1=O | *Streptomyces* sp. CL190 | Antioxidant | N/I | [53] |
| **93** | Naphterpin | [H][C@@]12C=C(C)CC[C@]1([H])C(C)(C)OC1=C2C(=O)C2=C(C(O)=C(C)C(O)=C2)C1=O | *Streptomyces* sp. CL190 | Antioxidant | N/I | [54] |
| **94** | 6,7-dihydroxy-3,4-dihydroiso quinoline- 3-carboxylic acid | OC(=O)C1CC2=CC(=O)C(O)=CC2=CN1 | *Streptomyces* sp. 8812 | Antioxidant | Terrestrial (Free-living) | [55] |
| **95** | 6-Acetylphenazine-1-carboxylic acid methyl ester (3) | COC(=O)C1=CC=CC2=NC3=C(C=CC=C3N=C12)C(C)=O | *Streptomyces* sp. 833 | Antioxidant | Terrestrial (Free-living) | [56] |
| **96** | methyl saphenate (4) | COC(=O)C1=CC=CC2=NC3=C(C=CC=C3N=C12)C(C)O | *Streptomyces* sp. 833 | Antioxidant | Terrestrial (Free-living) | [56] |
| **97** | Phenazostatin A | COC(=O)C1=CC=CC2=NC3=C(C=CC=C3N=C12)C(C)OC1=CC2=NC3=CC=CC=C3N=C2C=C1 | *Streptomyces* sp. 833 | Antioxidant | Terrestrial (Free-living) | [56] |
| **98** | Phenazostatin B | COC(=O)C1=CC=CC2=NC3=C(C=CC=C3N=C12)C(C)C(C)C1=CC=CC2=NC3=C(C=CC=C3N=C12)C(=O)OC | *Streptomyces* sp. 833 | Antioxidant | Terrestrial (Free-living) | [56] |
| **99** | Phenazoviridin | CC1OC(OC(=O)C2=CC=CC3=NC4=C(CC=C(C)C)C=CC=C4N=C23)C(O)C(O)C1O | *Streptomyces* sp. HR04 | Antioxidant | Terrestrial (Free-living) | [57] |
| **100** | Tetrangomycin | C[C@]1(O)CC(=O)C2=C(C1)C=CC1=C2C(=O)C2=C(C(O)=CC=C2)C1=O | *Streptomyces* sp. CAH29 | Antioxidant | Terrestrial (Free-living) | [58] |
| **101** | a-hydroxyacetovanillone (3) | COC1=C(O)C=CC(=C1)C(=O)CO | *Streptomyces* sp. YIM66017 | Antioxidant | Terrestrial (Symbiont) | [59] |
| **102** | cyclo(Gly-Trp) (4) | [H][C@@]1(CC2=CNC3=C2C=CC=C3)NC(=O)CNC1=O | *Streptomyces* sp. YIM66017 | Antioxidant | Terrestrial (Symbiont) | [59] |
| **103** | Herbimycin A^b^ | COC1CC(C)C(OC)C2=CC(=O)C=C(NC(=O)\C(C)=C\C=C/C(OC)C(OC(N)=O)\C(C)=C\C(C)C1OC)C2=O | *Streptomyces* sp. AO-0511 | Antioxidant | Terrestrial (Free-living) | [60] |
| **104** | Dihydroherbimycin A^b^ | COC1CC(C)C(OC)C2=C(O)C(NC(=O)\C(C)=C\C=C/C(OC)C(OC(N)=O)\C(C)=C\C(C)C1OC)=CC(O)=C2 | *Streptomyces* sp. AO-0511 | Antioxidant | Terrestrial (Free-living) | [60] |
| **105** | Mycotrienin I^b^ | COC1CC(=O)NC2=CC(=O)C=C(CC\C=C(C)/C(O)C(C)C(C\C=C\C=C\C=C\1)OC(=O)C(C)NC(=O)C1CCCCC1)C2=O | *Streptomyces* sp. USF-319 | Antioxidant | Terrestrial (Free-living) | [61] |
| **106** | Trienomycin A^b^ | COC1CC(=O)NC2=CC(CC\C=C(C)/C(O)C(C)C(C\C=C\C=C\C=C\1)OC(=O)C(C)NC(=O)C1CCCCC1)=CC(O)=C2 | *Streptomyces* sp. USF-319 | Antioxidant | Terrestrial (Free-living) | [61] |
| **107** | Trienomycin B | COC1CC(=O)NC2=CC(CC\C=C(C)/C(O)C(C)C(C\C=C\C=C\C=C\1)OC(=O)C(C)NC(=O)CC(C)C)=CC(O)=C2 | *Streptomyces* sp. USF-319 | Antioxidant | Terrestrial (Free-living) | [61] |
| **108** | Mycotrienin II^b^ | COC1CC(=O)NC2=C(O)C(CC\C=C(C)/C(O)C(C)C(C\C=C\C=C\C=C\1)OC(=O)C(C)NC(=O)C1CCCCC1)=CC(O)=C2 | *Streptomyces* sp. USF-319 | Antioxidant | Terrestrial (Free-living) | [61] |
| **109** | 6-hydroxy-7-methoxycoumarin^b^ | COC1=C(O)C=C2C=CC(=O)OC2=C1 | *Streptomyces* sp. TC1 | Antioxidant | Terrestrial (Free-living) | [62] |
| **110** | guai-2-en-10a-ol^b^ | CC1=C2C=CCCC(C)(O)C2CC1 | *Streptomyces* sp. ZJG1 | Antioxidant | Marine (Symbiont) | [63] |
| **111** | Carazostatin (1) | CCCCCCCC1=C(C)C(O)=CC2=C1NC1=C2C=CC=C1 | *S. chromofuscus* DC118 | Antioxidant | Terrestrial (Free-living) | [64] |
| **112** | Carbazomycin B (5) | COC1=C(O)C2=C(NC3=C2C=CC=C3)C(C)=C1C | *Streptomyces* sp. | Antioxidant | Terrestrial (Free-living) | [64] |
| **113** | cyclo(L-Phe-L-NMe-Tyr) (1) | CN1[C@@H](CC2=CC=C(O)C=C2)C(=O)N[C@@H](CC2=CC=CC=C2)C1=O | *Streptomyces* sp. SC0581 | Antioxidant | Terrestrial (Free-living) | [65] |
| **114** | cyclo(L-Phe-L-NMe-DOPA) (2) | CN1[C@@H](CC2=CC(O)=C(O)C=C2)C(=O)N[C@@H](CC2=CC=CC=C2)C1=O | *Streptomyces* sp. SC0581 | Antioxidant | Terrestrial (Free-living) | [65] |
| **115** | cyclo[L-Phe-L-(NMe-3-(NMe-3-O-α-L-rhamnopyranosyl)- DOPA] (3) | CC1O[C@@H](OC2=C(O)C=CC(C[C@@H]3N(C)C(=O)[C@H](CC4=CC=CC=C4)NC3=O)=C2)[C@@H](O)C(O)[C@H]1O | *Streptomyces* sp. SC0581 | Antioxidant | Terrestrial (Free-living) | [65] |
| **116** | 5-(2,4-dimethylbenzyl)pyrrolidin-2-one (DMBPO)^b^ | CC1=CC(C)=C(CC2CCC(=O)N2)C=C1 | *Streptomyces* VITSVK5 spp. | Antioxidant | Marine (Free-living) | [66] |
| **117** | 2-Allyloxyphenol | OC1=C(OCC=C)C=CC=C1 | *Streptomyces* sp. MS1/7 | Antioxidant | Marine (Free-living) | [67] |
| **118** | Hydroxy marilone C | COC1=C(C)C(OC\C=C(/C)CCC=C(C)C)=CC2=C1C(O)OC2=O | *S. badius* M7 | Antioxidant | Terrestrial (Free-living) | [68] |
| **119** | JBIR-85 | [H][C@@]12C[C@@H](C(C)=O)[C@@]34C[C@]1([H])[C@@](OC)(C(=O)O2)C(O)=C3C(=O)C1=C(C(O)=C(OC)C=C1O)C4=O | *Streptomyces* sp. RI-77 | Antioxidant | Terrestrial (Free-living) | [69] |
| **120** | 3-(3,5-Di-Tert-Butyl-4-Fluorophenyl)Propanoic Acid | CC(C)(C)C1=CC(CCC(O)=O)=CC(=C1F)C(C)(C)C | *Streptomyces* sp. TC1 | Antioxidant | Terrestrial (Free-living) | [70] |
| **121** | 2-(1,1-Diallylbut-3-Enyl)-5-Nonylphenol | CCCCCCCCCC1=CC=C(C(O)=C1)C(CC=C)(CC=C)CC=C | *Streptomyces* sp. TC1 | Antioxidant | Terrestrial (Free-living) | [70] |
| **122** | CHEMBL3104963 | CC(C)C[C@H](NC(=O)[C@@H](O)C[C@H](N)CN)C(N)=O | *Streptomyces* sp. TC1 | Antioxidant | Terrestrial (Free-living) | [70] |
| **123** | JBIR-94 | COC1=CC(CCC(=O)NCCCCNC(=O)CCC2=CC(OC)=C(O)C=C2)=CC=C1O | *Streptomyces* sp. R56-07 | Antioxidant | Terrestrial (Free-living) | [71] |
| **124** | JBIR-125 | CC(=O)NCCCCN(CCCNC(=O)CCC1=CC=C(O)C=C1)C(=O)CCC1=CC=C(O)C=C1 | *Streptomyces* sp. R56-07 | Antioxidant | Terrestrial (Free-living) | [71] |
| **125** | 12T061A | CC(OC(C)=O)C1C2=C(O)C3=CC=CC(O)=C3C(O)=C2C(=O)CC1(C)O | *Streptomyces* sp. 12T061 | Antioxidant | Terrestrial (Free-living) | [72] |
| **126** | 12T061C | COC1=C2C(=CC=C1O)C=C1C(C(C)OC(C)=O)C(C)(O)CC(=O)C1=C2O | *Streptomyces* sp. 12T061 | Antioxidant | Terrestrial (Free-living) | [72] |
| **127** | 10T024A | OC(=O)CN1C2=CC=CC=C2NC2=C1C=CC=C2C(O)=O | *Streptomyces* sp. 10T024 | Antioxidant | N/I | [73] |
| **128** | 1-hydroxy-1-norresistomycin | CC1=C2C(O)=CC3=C4C2=C(C(O)=C1)C(=O)C1=C4C(=C(O)C=C1O)C(=O)C3(C)O | *S. variabilis* | Antioxidant | Marine (Symbiont) | [74] |
| **129** | 3′-hydroxy-5-methoxy-3,4-methylenedioxybiphenyl (1) | COC1=CC(=CC2=C1OCO2)C1=CC(O)=CC=C1 | *Streptomyces* sp. BO-07 | Antioxidant | Terrestrial (Symbiont) | [75] |
| **130** | 3′- hydroxy-5,5′-dimethoxy-3,4-methylenedioxybiphenyl (2) | COC1=CC(=CC(O)=C1)C1=CC2=C(OCO2)C(OC)=C1 | *Streptomyces* sp. BO-07 | Antioxidant | Terrestrial (Symbiont) | [75] |
| **131** | Squamocin (1) | CCCCCC[C@H](O)CCC[C@H](O)[C@H]1CC[C@@H](O1)[C@H]1CC[C@@H](O1)[C@H](O)CCCCCCCCCCCCC1=C[C@H](C)OC1=O | *Streptomyces* sp. VE2 | Antioxidant | Terrestrial (Symbiont) | [76] |
| **132** | Rollidecin B (2) | CCCCCCCCCC[C@H](O)[C@H](O)CC[C@@H](O)[C@H]1CC[C@H](O1)[C@H]1CC[C@H](CCCCCCC[C@@H](O)CC2=C[C@H](C)OC2=O)O1 | *Streptomyces* sp. VE2 | Antioxidant | Terrestrial (Symbiont) | [76] |
| **133** | 1"-O-methyl-8-hydroxymethyl-daidzein | COCC1=C(O)C=CC2=C1OC=C(C2=O)C1=CC=C(O)C=C1 | *Streptomyces* sp. YIM 65408 | Antioxidant | Terrestrial (Symbiont) | [77] |
| **134** | 2-amino-3,4-dihydroxy-5-methoxybenzamide | COC1=CC(C(N)=O)=C(N)C(O)=C1O | *Streptomyces* sp. YIM 67086 | Antioxidant | Terrestrial (Symbiont) | [78] |
| **135** | 2,6-dimethoxy terephthalic acid (1) | COC1=CC(=CC(OC)=C1C(O)=O)C(O)=O | *Streptomyces* sp. YIM66017 | Antioxidant | Terrestrial (Symbiont) | [59] |
| **136** | yangjinhualine A (2) | CC1=C(C(O)OC1=O)C1=CC=C(O)C=C1 | *Streptomyces* sp. YIM66017 | Antioxidant | Terrestrial (Symbiont) | [59] |
| **137** | Protocatechualdehyde | OC1=C(O)C=C(C=O)C=C1 | *S. lincolnensis* M-20 | Antioxidant | Terrestrial (Free-living) | [79,80] |
| **138** | Surugapyrone A | CC(C)C1=CC(O)=C(C)C(=O)O1 | *S. coelicoflavus* USF-6280 | Antioxidant | Terrestrial (Free-living) | [81] |
| **139** | Surugapyrrole A | [H]ON1C=C(C)C=C1C(=O)NCCC(=O)O[H] | *Streptomyces* sp. USF-6280 | Antioxidant | Terrestrial (Free-living) | [82] |
| **140** | Surugapyrrole B | [H]ON1C=C([H])C=C1C(=O)NCCC(=O)O[H] | *Streptomyces* sp. USF-6280 | Antioxidant | Terrestrial (Free-living) | [82] |
| **141** | 3,4-Dihydroisoquinoline-3-carboxylic Acid | OC(=O)C1CC2=CC(O)=C(O)C=C2C=N1 | *Streptomyces* sp. 8812 | Antioxidant | Terrestrial (Free-living) | [83] |

N/I: strain origin not informed in source article. ^a^Simplified Molecular-Input Line-Entry System (SMILES) is a line notation for chemical structure. The notation uses the American Standard Code for Information Interchange (ASCII) character encoding [84]. ^b^The gray highlighted compounds (i.e., **103**, **104**, **105**, **106**, **108**, **109**, **110**, and **116**) indicates not active metabolites or that they did not achieve more than 50% inhibition at the highest concentration evaluated in the DPPH radical scavenging assay. These compounds were included in the SALI analysis.

**References**

1. Ai, W.; Lin, X.P.; Tu, Z.; Tian, X.P.; Lu, X.; Mangaladoss, F.; Zhong, Z.L.; Liu, Y. Axinelline A, a new COX-2 inhibitor from Streptomyces axinellae SCSIO02208. *Nat. Prod. Res.* **2014**, *28*, 1219–1224, doi:10.1080/14786419.2014.891204.

2. Ali, A.; Khajuria, A.; Sidiq, T.; Kumar, A.; Thakur, N.L.; Naik, D.; Vishwakarma, R.A. Modulation of LPS induced inflammatory response by Lawsonyl monocyclic terpene from the marine derived Streptomyces sp. *Immunol. Lett.* **2013**, *150*, 79–86, doi:10.1016/j.imlet.2012.09.001.

3. Bae, M.; Park, S.H.; Kwon, Y.; Lee, S.K.; Shin, J.; Nam, J.W.; Oh, D.C. QM-HiFSA-aided structure determination of succinilenes A-D, new triene polyols from a marine-derived Streptomyces sp. *Mar. Drugs* **2017**, *15*, 1–14, doi:10.3390/md15020038.

4. Deng, H.; Zhang, N.; Wang, Y.; Chen, J.; Shen, J.; Wang, Z.; Xu, R.; Zhang, J.; Song, D.; Li, D. S632A3, a new glutarimide antibiotic, suppresses lipopolysaccharide-induced pro-inflammatory responses via inhibiting the activation of glycogen synthase kinase 3Β. *Exp. Cell Res.* **2012**, *318*, 2592–2603, doi:10.1016/j.yexcr.2012.08.008.

5. Takeiri, M.; Ota, E.; Nishiyama, S.; Kiyota, H.; Umezawa, K. Structure-activity relationship of 9-methylstreptimidone, a compound that induces apoptosis selectively in adult T-cell leukemia cells. *Oncol. Res.* **2012**, *20*, 7–14, doi:10.3727/096504012X13425470196056.

6. Ding, R.; Tang, J.; Gao, H.; Li, T.; Zhou, H.; Liu, L.; Yao, X.S. New methymycin derivatives of Streptomyces venezuelae ATCC 15439 and their inhibitory effects on human T cell proliferation mediated by PMA/ionomycin. *Arch. Pharm. Res.* **2012**, *35*, 1567–1572, doi:10.1007/s12272-012-0907-z.

7. Hassan, H.M.; Boonlarppradab, C.; Fenical, W. Actinoquinolines A and B, anti-inflammatory quinoline alkaloids from a marine-derived Streptomyces sp., strain CNP975. *J. Antibiot. (Tokyo).* **2016**, *69*, 511–514, doi:10.1038/ja.2016.56.

8. Hong, S.H.; Ban, Y.H.; Byun, W.S.; Kim, D.; Jang, Y.J.; An, J.S.; Shin, B.; Lee, S.K.; Shin, J.; Yoon, Y.J.; et al. Camporidines A and B: Antimetastatic and Anti-inflammatory Polyketide Alkaloids from a Gut Bacterium of Camponotus kiusiuensis. *J. Nat. Prod.* **2019**, *82*, 903–910, doi:10.1021/acs.jnatprod.8b01000.

9. Lee, J.; Kim, H.; Lee, T.G.; Yang, I.; Won, D.H.; Choi, H.; Nam, S.J.; Kang, H. Anmindenols A and B, inducible nitric oxide synthase inhibitors from a marine-derived Streptomyces sp. *J. Nat. Prod.* **2014**, *77*, 1528–1531, doi:10.1021/np500285a.

10. Lee, H.S.; An, B.J.; Kim, H.J.; Cho, Y.H.; Kim, D.I.; Jang, J.Y.; Kwak, J.H.; Lee, H.S.; Lee, Y.J.; Lee, J.S.; et al. Anti-inflammatory effect of violapyrones B and C from a marine-derived Streptomyces sp. *Nat. Prod. Sci.* **2015**, *21*, 251–254, doi:10.20307/nps.2015.21.4.251.

11. Lee, D.S.; Yoon, C.S.; Jung, Y.T.; Yoon, J.H.; Kim, Y.C.; Oh, H. Marine-Derived Secondary Metabolite, Griseusrazin A, Suppresses Inflammation through Heme Oxygenase-1 Induction in Activated RAW264.7 Macrophages. *J. Nat. Prod.* **2016**, *79*, 1105–1111, doi:10.1021/acs.jnatprod.6b00009.

12. Li, H.; Huang, H.; Hou, L.; Ju, J.; Li, W. Discovery of antimycin-type depsipeptides from a wbl gene mutant strain of deepsea-derived Streptomyces somaliensis SCSIO ZH66 and their effects on pro-inflammatory cytokine production. *Front. Microbiol.* **2017**, *8*, 1–8, doi:10.3389/fmicb.2017.00678.

13. Usuki, Y.; Ishii, S.; Ijiri, M.; Yoshida, K.I.; Satoh, T.; Horigome, S.; Yoshida, I.; Mishima, T.; Fujita, K.I. Evaluation of Inhibitory Activities of UK-2A, an Antimycin-Type Antibiotic, and Its Synthetic Analogues against the Production of Anti-inflammatory Cytokine IL-4. *J. Nat. Prod.* **2018**, *81*, 2590–2594, doi:10.1021/acs.jnatprod.8b00559.

14. Strangman, W.K.; Kwon, H.C.; Broide, D.; Jensen, P.R.; Fenical, W. Potent inhibitors of pro-inflammatory cytokine production produced by a marine-derived bacterium. *J. Med. Chem.* **2009**, *52*, 2317–2327, doi:10.1021/jm801110j.

15. Ma, J.; Lei, H.; Chen, X.; Bi, X.; Jiang, Y.; Han, L.; Huang, X. New anti-inflammatory metabolites produced by Streptomyces violaceoruber isolated from Equus burchelli feces. *J. Antibiot. (Tokyo).* **2017**, *70*, 991–994, doi:10.1038/ja.2017.75.

16. Ma, J.; Cao, B.; Liu, C.; Guan, P.; Mu, Y.; Jiang, Y.; Han, L.; Huang, X. Actinofuranones D-I from a lichen-associated actinomycetes, streptomyces gramineus, and their anti-inflammatory effects. *Molecules* **2018**, *23*, doi:10.3390/molecules23092393.

17. Ma, J.; Cao, B.; Chen, X.; Xu, M.; Bi, X.; Guan, P.; Jiang, Y.; Xu, J.; Han, L.; Huang, X. Violacin A, a new chromanone produced by Streptomyces violaceoruber and its anti-inflammatory activity. *Bioorganic Med. Chem. Lett.* **2018**, *28*, 947–951, doi:10.1016/j.bmcl.2018.01.051.

18. Nalli, Y.; Gupta, S.; Khajuria, V.; Singh, V.P.; Sajgotra, M.; Ahmed, Z.; Thakur, N.L.; Ali, A. TNF-α and IL-6 inhibitory effects of cyclic dipeptides isolated from marine bacteria Streptomyces sp. *Med. Chem. Res.* **2017**, *26*, 93–100, doi:10.1007/s00044-016-1730-8.

19. Petříčková, K.; Pospíšil, S.; Kuzma, M.; Tylová, T.; Jágr, M.; Tomek, P.; Chroňáková, A.; Brabcová, E.; Anděra, L.; Krištůfek, V.; et al. Biosynthesis of Colabomycin E, a New Manumycin-Family Metabolite, Involves an Unusual Chain-Length Factor. *ChemBioChem* **2014**, *15*, 1334–1345, doi:10.1002/cbic.201400068.

20. Yang, X.W.; Peng, K.; Liu, Z.; Zhang, G.Y.; Li, J.; Wang, N.; Steinmetz, A.; Liu, Y. Strepsesquitriol, a rearranged zizaane-type sesquiterpenoid from the deep-sea-derived actinomycete streptomyces sp. SCSIO 10355. *J. Nat. Prod.* **2013**, *76*, 2360–2363, doi:10.1021/np400923c.

21. Graziani, E.I.; Ritacco, F. V.; Bernan, V.S.; Telliez, J.-B. Phaeochromycins A−E, Anti-inflammatory Polyketides Isolated from the Soil Actinomycete Streptomyces p haeochromogenes LL-P018. *J. Nat. Prod.* **2005**, *68*, 1262–1265, doi:10.1021/np0500629.

22. Gullo, V.; Conover, M.; Cooper, R.; Federbush, C.; Horan, A.C.; Kung, T.; Marquez, J.; Patel, M.; Watnick, A. Sch 36605, a novel anti-inflammatory compound. Taxonomy, fermentation, isolation and biological properties. *J. Antibiot. (Tokyo).* **1988**, *41*, 20–24, doi:10.7164/antibiotics.41.20.

23. Kuriyama, K.; Fujiwara, A.; Inagaki, K.; Abe, Y. Anti-inflammatory action of a novel peptide, SEK-1005, isolated from a Streptomyces. *Eur. J. Pharmacol.* **2000**, *390*, 223–228, doi:10.1016/S0014-2999(00)00017-0.

24. Kino, T.; Hatanaka, H.; Hashimoto, M.; Nishiyama, M.; Goto, T.; Okuhara, M.; Kohsaka, M.; Aoki, H.; Imanaka, H. FK-506, a novel immunosuppressant isolated from a Streptomyces. I. Fermentation, isolation, and physico-chemical and biological characteristics. *J. Antibiot. (Tokyo).* **1987**, *40*, 1249–1255, doi:10.7164/antibiotics.40.1249.

25. Kino, T.; Hatanaka, H.; Miyata, S.; Inamura, N.; Nishiyama, M.; Yajima, T.; Goto, T.; Okuhara, M.; Kohsaka, M.; Aoki, H.; et al. Fk-506, A Novel Immunosuppressant Isolated From A Streptomyces II. Immunosuppressive Effect Of Fk-506 In Vitro. *J. Antibiot. (Tokyo).* **1987**, *40*, 1256–1265, doi:10.7164/antibiotics.40.1256.

26. Pereira, R.; Santos Medeiros, Y.; Fröde, T.S. Antiinflammatory effects of Tacrolimus in a mouse model of pleurisy. *Transpl. Immunol.* **2006**, *16*, 105–111, doi:10.1016/j.trim.2006.04.001.

27. Vigil, S.V.G.; de Liz, R.; Medeiros, Y.S.; Fröde, T.S. Efficacy of tacrolimus in inhibiting inflammation caused by carrageenan in a murine model of air pouch. *Transpl. Immunol.* **2008**, *19*, 25–29, doi:10.1016/j.trim.2008.01.003.

28. Lee, S.J.; Kim, H.P.; Park, B.K.; Ahn, S.C.; Lee, H.S.; Ahn, J.S. Topical anti-inflammatory activity of dianemycin isolated from Streptomyces sp. MT 2705-4. *Arch. Pharm. Res.* **1997**, *20*, 372–374, doi:10.1007/BF02976203.

29. Mori, A.; Kaminuma, O.; Ogawa, K.; Nakata, A.; Egan, R.W.; Akiyama, K.; Okudaira, H. Control of IL-5 production by human helper T cells as a treatment for eosinophilic inflammation: Comparison of in vitro and in vivo effects between selective and nonselective cytokine synthesis inhibitors. *J. Allergy Clin. Immunol.* **2000**, *106*, S58–S64, doi:10.1067/mai.2000.106775.

30. Nakayama, O.; Yagi, M.; Tanaka, M.; Kiyoto, S.; Uchida, I.; Hashimoto, M.; Okuhara, M.; Kohsaka, M. WS-7528, a new isoflavanone with estrogen activity isolated from streptomyces sp. No. 7528. Taxonomy, fermentation, isolation, physico-chemical properties and biological activities. *J. Antibiot. (Tokyo).* **1990**, *43*, 1394–1402, doi:10.7164/antibiotics.43.1394.

31. Taechowisan, T.; Lu, C.; Shen, Y.; Lumyong, S. Anti-inflammatory effects of 4-arylcoumarins in LPS-induced murine macrophage RAW 264.7 cells. *Pharm. Biol.* **2006**, *44*, 576–580, doi:10.1080/13880200600896694.

32. Taechowisan, T.; Wanbanjob, A.; Tuntiwachwuttikul, P.; Liu, J. Anti-inflammatory activity of lansais from endophytic Streptomyces sp. SUC1 in LPS-induced RAW 264.7 cells. *Food Agric. Immunol.* **2009**, *20*, 67–77, doi:10.1080/09540100902730064.

33. Taechowisan, T.; Wanbanjob, A.; Tuntiwachwuttikul, P.; Liu, J. Anti-inflammatory effects of lansai C and D cause inhibition of STAT-1 and NF-κB activations in LPS-induced RAW 264.7 cells. *Food Agric. Immunol.* **2010**, *21*, 57–64, doi:10.1080/09540100903419592.

34. Park, E.J.; Pezzuto, J.M.; Jang, K.H.; Nam, S.J.; Bucarey, S.A.; Fenical, W. Suppression of nitric oxide synthase by thienodolin in lipopolysaccharide- stimulated RAW 264.7 murine macrophage cells. *Nat. Prod. Commun.* **2012**, *7*, 789–794, doi:10.1177/1934578x1200700625.

35. Trischman, J.A.; Tapiolas, D.M.; Jensen, P.R.; Dwight, R.; Fenical, W.; McKee, T.C.; Ireland, C.M.; Stout, T.J.; Clardy, J. Salinamides A and B: anti-inflammatory depsipeptides from a marine streptomycete. *J. Am. Chem. Soc.* **1994**, *116*, 757–758, doi:10.1021/ja00081a042.

36. Renner, M.K.; Shen, Y.C.; Cheng, X.C.; Jensen, P.R.; Frankmoelle, W.; Kauffman, C.A.; Fenical, W.; Lobkovsky, E.; Clardy, J. Cyclomarins A-C, new antiinflammatory cyclic peptides produced by a marine bacterium (Streptomyces sp.). *J. Am. Chem. Soc.* 1999, *121*, 11273–11276.

37. Alvarino, R.; Alonso, E.; Lacret, R.; Oves-Costales, D.; Genilloud, O.; Reyes, F.; Alfonso, A.; Botana, L.M. Caniferolide A, a Macrolide from Streptomyces caniferus, Attenuates Neuroinflammation, Oxidative Stress, Amyloid-Beta, and Tau Pathology in Vitro. *Mol. Pharm.* **2019**, *16*, 1456–1466, doi:10.1021/acs.molpharmaceut.8b01090.

38. Kondratyuk, T.P.; Park, E.J.; Yu, R.; Van Breemen, R.B.; Asolkar, R.N.; Murphy, B.T.; Fenical, W.; Pezzuto, J.M. Novel marine phenazines as potential cancer chemopreventive and anti-inflammatory agents. *Mar. Drugs* **2012**, *10*, 451–464, doi:10.3390/md10020451.

39. Raghava Rao, K.V.; Mani, P.; Satyanarayana, B.; Raghava Rao, T. Purification and structural elucidation of three bioactive compounds isolated from Streptomyces coelicoflavus BC 01 and their biological activity. *3 Biotech* **2017**, *7*, doi:10.1007/s13205-016-0581-9.

40. Stankovic, N.; Radulovic, V.; Petkovic, M.; Vuckovic, I.; Jadranin, M.; Vasiljevic, B.; Nikodinovic-Runic, J. Streptomyces sp. JS520 produces exceptionally high quantities of undecylprodigiosin with antibacterial, antioxidative, and UV-protective properties. *Appl. Microbiol. Biotechnol.* **2012**, *96*, 1217–1231, doi:10.1007/s00253-012-4237-3.

41. Naine, S.J.; Devi, C.S.; Mohanasrinivasan, V.; Doss, C.G.P.; Kumar, D.T. Binding and molecular dynamic studies of sesquiterpenes (2R-acetoxymethyl-1,3,3-trimethyl-4t-(3-methyl-2-buten-1-yl)-1t-cyclohexanol) derived from marine Streptomyces sp. VITJS8 as potential anticancer agent. *Appl. Microbiol. Biotechnol.* **2016**, *100*, 2869–2882, doi:10.1007/s00253-015-7156-2.

42. Sajjad, W.; Ahmad, S.; Aziz, I.; Azam, S.S.; Hasan, F.; Shah, A.A. Antiproliferative, antioxidant and binding mechanism analysis of prodigiosin from newly isolated radio-resistant Streptomyces sp. strain WMA-LM31. *Mol. Biol. Rep.* **2018**, *45*, 1787–1798, doi:10.1007/s11033-018-4324-3.

43. Leirós, M.; Alonso, E.; Sanchez, J.A.; Rateb, M.E.; Ebel, R.; Houssen, W.E.; Jaspars, M.; Alfonso, A.; Botana, L.M. Mitigation of ROS insults by streptomyces secondary metabolites in primary cortical neurons. *ACS Chem. Neurosci.* **2014**, *5*, 71–80, doi:10.1021/cn4001878.

44. Cheng, C.; Othman, E.M.; Reimer, A.; Grüne, M.; Kozjak-Pavlovic, V.; Stopper, H.; Hentschel, U.; Abdelmohsen, U.R. Ageloline A, new antioxidant and antichlamydial quinolone from the marine sponge-derived bacterium Streptomyces sp. SBT345. *Tetrahedron Lett.* **2016**, *57*, 2786–2789, doi:10.1016/j.tetlet.2016.05.042.

45. Hori, R.; Yamaguchi, K.; Sato, H.; Watanabe, M.; Tsutsumi, K.; Iwamoto, S.; Abe, M.; Onodera, H.; Nakamura, S.; Nakai, R. The discovery and characterization of K-563, a novel inhibitor of the Keap1/Nrf2 pathway produced by Streptomyces sp. *Cancer Med.* **2019**, *8*, 1157–1168, doi:10.1002/cam4.1949.

46. Jiménez, J.T.; Sturdíková, M.; Brezová, V.; Svajdlenka, E.; Novotová, M. Screening of mutant strain Streptomyces mediolani sp. AC37 for (-)-8-O-methyltetrangomycin production enhancement. *J. Microbiol.* **2012**, *50*, 1014–1023, doi:10.1007/s12275-012-2025-5.

47. Kim, K.-Y.; Lee, S.-G.; Baek, S.Y.; Lee, E.H.; Jang, E.J.; Lee, J.-H.; Ahn, S.-C.; Chang, J.-H.; Oh, T.W.; Kim, S.-H.; et al. Salinomycin ameliorates oxidative hepatic damage through AMP-activated protein kinase, facilitating autophagy. *Toxicol. Appl. Pharmacol.* **2018**, *360*, 141–149, doi:10.1016/j.taap.2018.10.002.

48. Kato, S.; Shindo, K.; Kawai, H.; Odagawa, A.; Matsuoka, M.; Mochizuki, J. Pyrrolostatin, a novel lipid peroxidation inhibitor from Streptomyces chrestomyceticus. Taxonomy, fermentation, isolation, structure elucidation and biological properties. *J. Antibiot. (Tokyo).* **1993**, *46*, 892–899, doi:10.7164/antibiotics.46.892.

49. Kim, W.; Kim, J.; Kim, C.-J.; Lee, K.-H.; Yoo, I.-D. Benzastatins A, B, C, and D: New Free Radical Scavengers from Streptomyces nitrosporeus 30643. I. Taxonomy, Fermentation, Isolation, Physico-chemical Properties and Biological Activities. *J. Antibiot. (Tokyo).* **1996**, *49*, 20–25, doi:10.7164/antibiotics.49.20.

50. Komiyama, K.; Funayama, S.; Anraku, Y.; Mita, A.; Takahashi, Y.; Omura, S. Isolation of isoflavonoids possessing antioxidant activity from the fermentation broth of Streptomyces sp. *J. Antibiot. (Tokyo).* **1989**, *42*, 1344–1349, doi:10.7164/antibiotics.42.1344.

51. Shin-ya, K.; Furihata, K.; Teshima, Y.; Hayakawa, Y.; Seto, H. Structures of stealthins A and B, new free radical scavengers of microbial origin. *Tetrahedron Lett.* **1992**, *33*, 7025–7028, doi:10.1016/S0040-4039(00)60923-1.

52. Shin-ya, K.; Tanaka, M.; Furihata, K.; Hayakawa, Y.; Seto, H. Structure of carquinostatin a, a new neuronal cell protecting substance produced by Streptomyces exfoliatus. *Tetrahedron Lett.* **1993**, *34*, 4943–4944, doi:10.1016/S0040-4039(00)74052-4.

53. Takagi, H.; Motohashi, K.; Miyamoto, T.; Shin-ya, K.; Furihata, K.; Seto, H. Studies on terpenoids produced by actinomycetes isolation and structural elucidation of antioxidative agents, naphterpins B and C. *J. Antibiot. (Tokyo).* **2005**, *58*, 275–278, doi:10.1038/ja.2005.33.

54. Shin-Ya, K.; Imai, S.; Furihata, K.; Hayakawa, Y.; Kato, Y.; Vanduyne, G.D.; Clardy, J.; Seto, H. Isolation and structural elucidation of an antioxidative agent, naphterpin. *J. Antibiot. (Tokyo).* **1990**, *43*, 444–447, doi:10.7164/antibiotics.43.444.

55. Guśpiel, A.; Ziemska, J.; Cześcik, A.; Kawecki, R.; Solecka, J. Intracellular antioxidant activity of a streptomyces sp. 8812 secondary metabolite, 6,7-dihydroxy-3,4-dihydroisoquinoline-3-carboxylic acid, and its synthetic derivatives. *Acta Pol. Pharm. - Drug Res.* **2016**, *73*, 645–651.

56. Kim, W.G.; Ryoo, I.J.; Yun, B.S.; Shin-ya, K.; Seto, H.; Yoo, I.D. New diphenazines with neuronal cell protecting activity, phenazostatins A and B, produced by Streptomyces sp. *J. Antibiot. (Tokyo).* **1997**, *50*, 715–721, doi:10.7164/antibiotics.50.715.

57. Shinichiro, K.; Kazutoshi, S.; Yuji, Y.; Michiko, M.; Hiroyuki, K.; Junichiro, M. Phenazoviridin, a novel free radical scavenger from Streptomyces Sp. Taxonomy, fermentation, isolation, structure elucidation and biological properties. *J. Antibiot. (Tokyo).* **1993**, *46*, 1485–1493, doi:10.7164/antibiotics.46.1485.

58. Özakin, S.; Davis, R.W.; Umile, T.P.; Pirinccioglu, N.; Kizil, M.; Celik, G.; Sen, A.; Minbiole, K.P.C.; İnce, E. The isolation of tetrangomycin from terrestrial Streptomyces sp. CAH29: evaluation of antioxidant, anticancer, and anti-MRSA activity. *Med. Chem. Res.* **2016**, *25*, 2872–2881, doi:10.1007/s00044-016-1708-6.

59. Zhou, H.; Yang, Y.; Peng, T.; Li, W.; Zhao, L.; Xu, L.; Ding, Z. Metabolites of Streptomyces sp., an endophytic actinomycete from Alpinia oxyphylla. *Nat. Prod. Res.* **2014**, *28*, 265–267, doi:10.1080/14786419.2013.830219.

60. Chang, H.B.; Kim, J.H. Antioxidant properties of dihydroherbimycin A from a newly isolated Streptomyces sp. *Biotechnol. Lett.* **2007**, *29*, 599–603, doi:10.1007/s10529-006-9288-z.

61. Morimitsu, Y.; Hirota, A. Ansamycin Antibiotics as Free Radical Scavengers Isolated from Streptomyces by Using the Bactericidal Action of the Hydroxyl Radical. *Biosci. Biotechnol. Biochem.* **1996**, *60*, 1507–1509, doi:10.1271/bbb.60.1507.

62. Jaivel, N.; Rajesh, R.; Uvarani, C.; Marimuthu, P. In vitro antimicrobial evaluation of compound derived from streptomyces sp. TC1 against xanthomonas oryzae pv. Oryzae. *J. Pure Appl. Microbiol.* **2014**, *8*, 1299–1310.

63. Wen, L.; Chen, G.; Zhang, S.; You, T.; Liu, F.; Fu, Y.; Yao, X. In vitro antioxidant and acetylcholinesterase inhibitory activities of the sesquiterpenes of a symbiotic actinomycete Streptomyces sp. from South China sea. *Asian J. Chem.* **2013**, *25*, 6865–6869, doi:10.14233/ajchem.2013.14994.

64. Kato, S.; Kawasaki, T.; Urata, T.; Mochizuki, J. In vitro and ex vivo free radical scavenging activities of carazostatin, carbazomycin B and their derivatives. *J. Antibiot. (Tokyo).* **1993**, *46*, 1859–1865, doi:10.7164/antibiotics.46.1859.

65. Yang, L.; Mahal, A.; Liu, Y.; Li, H.; Wu, P.; Xue, J.; Xu, L.; Wei, X. Two new 2,5-diketopiperazines produced by Streptomyces sp. SC0581. *Phytochem. Lett.* **2017**, *20*, 89–92, doi:10.1016/j.phytol.2017.04.012.

66. Saurav, K.; Kannabiran, K. Cytotoxicity and antioxidant activity of 5-(2,4-dimethylbenzyl)pyrrolidin-2-one extracted from marine Streptomyces VITSVK5 spp. *Saudi J. Biol. Sci.* **2012**, *19*, 81–86, doi:10.1016/j.sjbs.2011.07.003.

67. Arumugam, M.; Mitra, A.; Jaisankar, P.; Dasgupta, S.; Sen, T.; Gachhui, R.; Kumar Mukhopadhyay, U.; Mukherjee, J. Isolation of an unusual metabolite 2-allyloxyphenol from a marine actinobacterium, its biological activities and applications. *Appl. Microbiol. Biotechnol.* **2010**, *86*, 109–117, doi:10.1007/s00253-009-2311-2.

68. El Sayed, O.H.; Asker, M.M.S.; Swelim, M.A.; Abbas, I.H.; Attwa, A.I.; El Awady, M.E. Production of hydroxy marilone C as a bioactive compound from Streptomyces badius. *J. Genet. Eng. Biotechnol.* **2016**, *14*, 161–168, doi:10.1016/j.jgeb.2016.04.001.

69. Izumikawa, M.; Satou, R.; Motohashi, K.; Nagai, A.; Ohnishi, Y.; Takagi, M.; Shin-Ya, K. Naphthoquinone-like polyketide isolated from Streptomyces sp. RI-77 and its predicted biosynthetic pathway. *J. Nat. Prod.* **2011**, *74*, 2588–2591, doi:10.1021/np200651e.

70. Jaivel, N.; Uvarani, C.; Rajesh, R.; Velmurugan, D.; Marimuthu, P. Natural occurrence of organofluorine and other constituents from streptomyces sp. TC1. *J. Nat. Prod.* **2014**, *77*, 2–8, doi:10.1021/np400360h.

71. Kawahara, T.; Izumikawa, M.; Otoguro, M.; Yamamura, H.; Hayakawa, M.; Takagi, M.; Shin-Ya, K. JBIR-94 and JBIR-125, antioxidative phenolic compounds from streptomyces sp. R56-07. *J. Nat. Prod.* **2012**, *75*, 107–110, doi:10.1021/np200734p.

72. Komoda, T.; Saeki, N.; Koseki, Y.; Kiyota, H. 12T061A and 12T061C, two new julichrome family compounds, as radical scavengers from Streptomyces sp. *J. Gen. Appl. Microbiol.* **2016**, *62*, 1–6, doi:10.2323/jgam.62.1.

73. Komoda, T.; Tamiya, Y.; Nishikawa, M. 10T024A, a New phenazine derivative, as a radical scavenger and a prostaglandin, leukotriene release suppressor. *Biosci. Biotechnol. Biochem.* **2011**, *75*, 2056–2058, doi:10.1271/bbb.110384.

74. Ramalingam, V.; Rajaram, R. Antioxidant activity of 1-hydroxy-1-norresistomycin derived from Streptomyces variabilis KP149559 and evaluation of its toxicity against zebra fish Danio rerio. *RSC Adv.* **2016**, *6*, 16615–16623, doi:10.1039/c5ra22558b.

75. Taechowisan, T.; Chaisaeng, S.; Phutdhawong, W.S. Antibacterial, antioxidant and anticancer activities of biphenyls from Streptomyces sp. BO-07: an endophyte in Boesenbergia rotunda (L.) Mansf A. *Food Agric. Immunol.* **2017**, *28*, 1330–1346, doi:10.1080/09540105.2017.1339669.

76. Taechowisan, T.; Singtotong, C.; Phutdhawong, W.S. Antibacterial and antioxidant activities of acetogenins from streptomyces sp. VE2; an endophyte in Vernonia cinerea (L.) less. *J. Appl. Pharm. Sci.* **2016**, *6*, 67–72, doi:10.7324/JAPS.2016.60810.

77. Yang, Y.; Yang, X.; Zhang, Y.; Zhou, H.; Zhang, J.; Xu, L.; Ding, Z. A new daidzein derivative from endophytic Streptomyces sp. YIM 65408. *Nat. Prod. Res.* 2013, *27*, 1727–1731.

78. Yang, X.; Peng, T.; Yang, Y.; Li, W.; Xiong, J.; Zhao, L.; Ding, Z. Antimicrobial and antioxidant activities of a new benzamide from endophytic Streptomyces sp. YIM 67086. *Nat. Prod. Res.* 2015, *29*, 331–335.

79. Kim, K.J.; Kim, M.A.; Jung, J.H. Antitumor and antioxidant activity of protocatechualdehyde produced from Streptomyces lincolnensis M-20. *Arch. Pharm. Res.* **2008**, *31*, 1572–1577, doi:10.1007/s12272-001-2153-7.

80. Kim, K.J.; Lee, J.H.; Yang, Y.J. Effect of interaction between protocatechualdehyde produced from streptomyces lincolnensis M-20 and copper ions on antioxidant and pro-oxidant activities. *Korean J. Microbiol.* **2014**, *50*, 22–26, doi:10.7845/kjm.2014.4007.

81. Sugiyama, Y.; Oya, A.; Kudo, T.; Hirota, A. Surugapyrone A from Streptomyces coelicoflavus strain usf-6280 as a new dpph radical-scavenger. *J. Antibiot. (Tokyo).* **2010**, *63*, 365–369, doi:10.1038/ja.2010.60.

82. Sugiyama, Y.; Watanabe, K.; Hirota, A. Surugapyrroles A and B, two new N-hydroxypyrroles, as DPPH radical-scavengers from Streptomyces sp. USF-6280 strain. *Biosci. Biotechnol. Biochem.* **2009**, *73*, 230–232, doi:10.1271/bbb.80617.

83. Solecka, J.; Gus̈piel, A.; Postek, M.; Ziemska, J.; Kawęcki, R.; Łęczycka, K.; Osior, A.; Pietrzak, B.; Pypowski, K.; Wyrzykowska, A. New derivatives of 3,4-dihydroisoquinoline-3-carboxylic acid with free-radical scavenging, d-amino acid oxidase, acetylcholinesterase and butyrylcholinesterase inhibitory activity. *Molecules* **2014**, *19*, 15866–15890, doi:10.3390/molecules191015866.

84. Weininger, D. SMILES, a chemical language and information system. 1. Introduction to methodology and encoding rules. *J. Chem. Inf. Model.* **1988**, *28*, 31–36, doi:10.1021/ci00057a005.
