# Supplementary material for: Streptomyces-Derived Metabolites with Potential Photoprotective Properties—A Systematic Literature Review and Meta-Analysis on the Reported Chemodiversity
Source: Molecules. 2020 Jul 15;25(14):3221. doi: 10.3390/molecules25143221 (PMC7397340; doi:10.3390/molecules25143221)
Supplement: Supplementary file 1 [file molecules-25-03221-s001.zip › Supplementary Materials/Table_S3.docx]

**Table S3.** *Streptomyces* strains source of bioactive crude extracts.

| **Source** | **Bioactivity** | **Strain name** | **Reference** |
| --- | --- | --- | --- |
| Terrestrial (Free-living) | Antioxidant | S. albus TRA-1 | [1] |
| Terrestrial (Free-living) | Antioxidant | S. cavouresis KU-V39 | [2] |
| Terrestrial (Free-living) | Antioxidant | S. cellulosae TES17 | [3] |
| Terrestrial (Free-living) | Antioxidant | S. cinnamonensis VITNS1 | [4] |
| Terrestrial (Free-living) | Antioxidant | S. colonosanans MUSC 93JT | [5] |
| Terrestrial (Free-living) | Antioxidant | S. fradiae VITIPMSB | [6] |
| Terrestrial (Free-living) | Antioxidant | S. griseus BW2-3 | [7] |
| Terrestrial (Free-living) | Antioxidant | S. sampsonii PA2 | [7] |
| Terrestrial (Free-living) | Antioxidant | S. tanashiensis KA2 | [7] |
| Terrestrial (Free-living) | Antioxidant | Streptomyces sp. NA2 | [7] |
| Terrestrial (Free-living) | Antioxidant | S. lavendulae SCA5 | [8] |
| Terrestrial (Free-living) | Antioxidant | S. lydicus A2 | [9] |
| Terrestrial (Free-living) | Antioxidant | S. nogalater NIIST A30 | [10] |
| Terrestrial (Free-living) | Antioxidant | Streptomyces sp. AC23 | [11] |
| Terrestrial (Free-living) | Antioxidant | Streptomyces sp. D25 | [12] |
| Terrestrial (Free-living) | Antioxidant | Streptomyces sp. Eri11 | [13] |
| Terrestrial (Free-living) | Antioxidant | Streptomyces sp. Loyola AR1 | [14] |
| Terrestrial (Free-living) | Antioxidant | Streptomyces sp. MJM 10778 | [15] |
| Terrestrial (Free-living) | Antioxidant | Streptomyces sp. OS-6 | [16] |
| Terrestrial (Free-living) | Antioxidant | Streptomyces sp. TES-25 | [16] |
| Terrestrial (Free-living) | Antioxidant | Streptomyces sp. PS4 | [17] |
| Terrestrial (Free-living) | Antioxidant | Streptomyces sp. SRDP-07 | [18] |
| Terrestrial (Free-living) | Antioxidant | Streptomyces sp. VITMSS05 | [19] |
| Terrestrial (Free-living) | Antioxidant | Streptomyces sp. VITNSJ2 | [20] |
| Terrestrial (Free-living) | Antioxidant | S. fradiae GOS1 | [21] |
| Terrestrial (Free-living) | Anti-inflammatory | S. fradiae GOS1 | [22] |
| Terrestrial (Free-living) | Antioxidant/Anti-inflammatory | Streptomyces sp. MJM 8637 | [23] |
| Terrestrial (Symbiont) | Antioxidant | S. albosporus J-8 | [24] |
| Terrestrial (Symbiont) | Antioxidant | S. globosus A012 | [25] |
| Terrestrial (Symbiont) | Antioxidant | S. hypolithicus A100 | [25] |
| Terrestrial (Symbiont) | Antioxidant | S. phaeochromogenes A009 | [25] |
| Terrestrial (Symbiont) | Antioxidant | Streptomyces sp. A071 | [25] |
| Terrestrial (Symbiont) | Antioxidant | S. hydrogenans NAF-1 | [26] |
| Terrestrial (Symbiont) | Antioxidant | Streptomyces sp. A0916 | [27] |
| Terrestrial (Symbiont) | Antioxidant | Streptomyces sp. IB 2014 I 73-1 | [28] |
| Terrestrial (Symbiont) | Antioxidant | Streptomyces sp. IB 2014 I 73-1HS | [28] |
| Terrestrial (Symbiont) | Antioxidant | Streptomyces sp. IB 2014 I 73-2HS | [28] |
| Terrestrial (Symbiont) | Antioxidant | Streptomyces sp. IB 2014 I 74-1 | [28] |
| Terrestrial (Symbiont) | Antioxidant | Streptomyces sp. IB 2014 I 74-2HS | [28] |
| Terrestrial (Symbiont) | Antioxidant | Streptomyces sp. IB 2014 I 74-3 | [28] |
| Terrestrial (Symbiont) | Antioxidant | Streptomyces sp. IB 2014 I 74-4 HS | [28] |
| Terrestrial (Symbiont) | Antioxidant | Streptomyces sp. IB 2014 I 74-7HS | [28] |
| Terrestrial (Symbiont) | Antioxidant | Streptomyces sp. IB 2014 I 75-1HS | [28] |
| Terrestrial (Symbiont) | Antioxidant | Streptomyces sp. IB 2014 I 75-2HS | [28] |
| Terrestrial (Symbiont) | Antioxidant | Streptomyces sp. IB 2014 I 75-4HS | [28] |
| Terrestrial (Symbiont) | Antioxidant | Streptomyces sp. IB 2014 I 77-1 | [28] |
| Terrestrial (Symbiont) | Antioxidant | Streptomyces sp. Loyola UGC | [29] |
| Terrestrial (Symbiont) | Antioxidant | Streptomyces sp. PnA 2 | [30] |
| Terrestrial (Symbiont) | Antioxidant | Streptomyces sp. PnA 3 | [30] |
| Terrestrial (Symbiont) | Antioxidant | Streptomyces sp. RT-18 | [31] |
| Terrestrial (Symbiont) | Antioxidant | Streptomyces sp. RT-50 | [31] |
| Terrestrial (Symbiont) | Antioxidant | Streptomyces sp. RT-56 | [31] |
| Terrestrial (Symbiont) | Antioxidant | Streptomyces sp. RT-67 | [31] |
| Terrestrial (Symbiont) | Antioxidant/Anti-inflammatory | S. flavoviridis A3WK | [32] |
| Marine (Free-living) | Antioxidant | S. antioxidans sp. nov. | [33] |
| Marine (Free-living) | Antioxidant | S. bluensi VSKB-3 | [34] |
| Marine (Free-living) | Antioxidant | S. carpaticus MK-01 | [35] |
| Marine (Free-living) | Antioxidant | S. coelicoflavus BC 01 | [36] |
| Marine (Free-living) | Antioxidant | S. coelicoflavus BC 02 | [36] |
| Marine (Free-living) | Antioxidant | S. coelicoflavus BC 04 | [36] |
| Marine (Free-living) | Antioxidant | S. griesoruber S2A | [37] |
| Marine (Free-living) | Antioxidant | S. laurentii VITMPS | [38] |
| Marine (Free-living) | Antioxidant | S. malaysiense sp. nov. | [39] |
| Marine (Free-living) | Antioxidant | S. mangrovisoli sp. nov. | [40] |
| Marine (Free-living) | Antioxidant | S. monashensis sp. nov. MUSC 1JT | [41] |
| Marine (Free-living) | Antioxidant | S. omiyaensis SCH2 | [42] |
| Marine (Free-living) | Antioxidant | S. parvulus VITJS11 | [43] |
| Marine (Free-living) | Antioxidant | S. pluripotens MUSC 137 | [44] |
| Marine (Free-living) | Antioxidant | S. spectabilis VITJS10 | [45] |
| Marine (Free-living) | Antioxidant | S. variabilis DV-35 | [46] |
| Marine (Free-living) | Antioxidant | Streptomyces sp. | [47] |
| Marine (Free-living) | Antioxidant | Streptomyces sp. LK3 | [48] |
| Marine (Free-living) | Antioxidant | Streptomyces sp. MUM212 | [49] |
| Marine (Free-living) | Antioxidant | Streptomyces sp. MUM256 | [50] |
| Marine (Free-living) | Antioxidant | Streptomyces sp. MUM265 | [51] |
| Marine (Free-living) | Antioxidant | Streptomyces sp. MUM292 | [52] |
| Marine (Free-living) | Antioxidant | Streptomyces sp. PM17 | [53] |
| Marine (Free-living) | Antioxidant | Streptomyces sp. SMS_7 | [54] |
| Marine (Free-living) | Antioxidant | Streptomyces sp. SMS_SU13 | [54] |
| Marine (Free-living) | Antioxidant | Streptomyces sp. SMS_SU21 | [54] |
| Marine (Free-living) | Antioxidant | Streptomyces sp. UTMC 1334 | [55] |
| Marine (Free-living) | Antioxidant | Streptomyces sp. VITJS4 | [56] |
| Marine (Free-living) | Antioxidant | Streptomyces sp. VITSD1 | [57] |
| Marine (Free-living) | Antioxidant/Anti-inflammatory | S. olivaceus MSU3 | [58] |
| Marine (Free-living) | Antioxidant/Anti-inflammatory | Streptomyces sp. VITPSA | [59] |
| Marine (Symbiont) | Antioxidant | Streptomyces sp. 23-2B | [60] |
| Marine (Symbiont) | Antioxidant | Streptomyces sp. SC 156 | [61] |
| NA | Antioxidant | Streptomyces sp. KB1 | [62] |
| NA | Antioxidant | Streptomyces sp. KB3 | [62] |
| NA | Anti-inflammatory | S. carpaticus | [63] |

**References**

1. Bhosale, H.; Bismile, P.; Kadam, T.; Shaheen, U. Antioxidant, enzyme inhibitory and antifungal activities of actinomycetes isolated from Curcuma longa rhizosphere. *Int. J. Pharm. Pharm. Sci.* **2016**, *8*, 307–311.

2. Narendhran, S.; Rajiv, P.; Vanathi, P.; Sivaraj, R. Spectroscopic analysis of bioactive compounds from Streptomyces Cavouresis KUV39: Evaluation of antioxidant and cytotoxicity activity. *Int. J. Pharm. Pharm. Sci.* **2014**, *6*, 319–322.

3. Rani, R.; Arora, S.; Kaur, J.; Manhas, R.K. Phenolic compounds as antioxidants and chemopreventive drugs from Streptomyces cellulosae strain TES17 isolated from rhizosphere of Camellia sinensis. *BMC Complement. Altern. Med.* **2018**, *18*, 1–15, doi:10.1186/s12906-018-2154-4.

4. Subathradevi, C.; Devi, P.; Jemimahnaine, S.; Mohanasrinivasan, V. Antibacterial and Antioxidant Property of Streptomyces cinnamonensis VITNS1 Isolated from Serkadu Region, Vellore, Tamil Nadu, India. *Anti-Infective Agents* **2014**, *12*, 206–212, doi:10.2174/2211352512666140714175749.

5. Law, J.W.F.; Ser, H.L.; Duangjai, A.; Saokaew, S.; Bukhari, S.I.; Khan, T.M.; Ab Mutalib, N.S.; Chan, K.G.; Goh, B.H.; Lee, L.H. Streptomyces colonosanans sp. nov., a novel actinobacterium isolated from Malaysia mangrove soil exhibiting antioxidative activity and cytotoxic potential against human colon cancer cell lines. *Front. Microbiol.* **2017**, *8*, 1–15, doi:10.3389/fmicb.2017.00877.

6. Chakraborty, I.; Redkar, P.; Munjal, M.; Sathish Kumar, S.R.; Bhaskara Rao, K. V. Isolation and characterization of pigment producing marine actinobacteria from mangrove soil and applications of bio-pigments. *Der Pharm. Lett.* **2015**, *7*, 93–100.

7. Parimala, G.S.A.; Manon, M. V; Karthiyaini, D.; Priyadharshini, U.; Jeeva, S.; Brindha, P. V Bioprospecting fungicidal metabolite producing marine actinomycetes from southern coastal regions of India. *Int. J. Life Sci. Pharma Res.* **2017**, *7*, 55–64.

8. Kumar, P.S.; Abdullah Al-Dhabi, N.; Duraipandiyan, V.; Balachandran, C.; Kumar, P.P.; Ignacimuthu, S. In vitro antimicrobial, antioxidant and cytotoxic properties of Streptomyces lavendulae strain SCA5. *BMC Microbiol.* **2014**, *14*, 291/1-291/26, 26 pp., doi:10.1186/s12866-014-0291-6.

9. Lertcanawanichakul, M.; Pondet, K.; Kwantep, J. In vitro antimicrobial and antioxidant activities of bioactive compounds (secondary metabolites) extracted from Streptomyces lydicus A2. *J. Appl. Pharm. Sci.* **2015**, *5*, 017–021, doi:10.7324/JAPS.2015.50204.

10. Jacob, J.; Rajendran, R.U.; Priya, S.H.; Purushothaman, J.; Saraswathy Amma, D.K.B.N. Enhanced antibacterial metabolite production through the application of statistical methodologies by a Streptomyces nogalater NIIST A30 isolated from Western Ghats forest soil. *PLoS One* **2017**, *12*, 1–21, doi:10.1371/journal.pone.0175919.

11. Subathra Devi, C.; Kumari, A.; Jain, N.; Naine S., J.; Mohanasrinivasan, V. Screening of actinomycetes isolated from soil samples for antibacterial and antioxidant activity. *Int. J. Pharm. Pharm. Sci.* **2013**, *5*, 483–489, 7 pp.

12. Radhakrishnan, M.; Gopikrishnan, V.; Vijayalakshmi, G.; Kumar, V. In vitro antioxidant activity and antimicrobial activity against biofilm forming bacteria by the pigment from Desert soil Streptomyces sp D25. *J. Appl. Pharm. Sci.* **2016**, *6*, 148–150, doi:10.7324/JAPS.2016.60626.

13. Kai, Z.; Xia Ling, G.; Zheng Jun, X.; Li Hua, L.; Rong Jun, C.X.J.; eng; Hong, G.; Kai, J.; Isomaro, Y. Isolation and characterization of a novel streptomyces strain Eri11 exhibiting antioxidant activity from the rhizosphere of Rhizoma Curcumae Longae. *African J. Microbiol. Res.* **2011**, *5*, 1291–1297, doi:10.5897/ajmr11.095.

14. Praveen Kumar, P.; Preetam Raj, J.P.; Nimal Christhudas, I.V.S.; Sagaya Jansi, R.; Narbert Raj, M.; Agastian, P. α-Glucosidase Inhibition and Antioxidant Properties of Streptomyces sp.: In Vitro. *Appl. Biochem. Biotechnol.* **2014**, *172*, 1687–1698, doi:10.1007/s12010-013-0650-z.

15. Lee, D.-R.; Lee, S.-K.; Choi, B.-K.; Cheng, J.; Lee, Y.-S.; Yang, S.H.; Suh, J.-W. Antioxidant activity and free radical scavenging activities of Streptomyces sp. strain MJM 10778. *Asian Pac. J. Trop. Med.* **2014**, *7*, 962–967.

16. Kaur, J.; Manhas, R.K.; Rani, R.; Arora, S. Actinobacteria from soil as potential free radical scavengers. *Malays. J. Microbiol.* **2013**, *13*, 217–227.

17. Geo, H.N.; Panneerselvam, A. Studies on bioactive potential of Streptomyces SPP. (KX710212). Isolated from environmental polluted sample, Ranipet, Vellore, India. *Asian J. Pharm. Clin. Res.* **2016**, *9*, 356–359, doi:10.22159/ajpcr.2016.v9s3.14788.

18. Prashith, K.T.R.; Dileep, N.; Syed, J.; Rakesh, K.N.; Sunita, C.M.; Onkarappa, R. Biological activities of Streptomyces species SRDP-07 isolated from soil of Thirthahalli, Karnataka, India. *Int. J. Drug Dev. Res.* **2013**, *5*, 268–285.

19. Revathy, T.; Jayasri, M.A.; Suthindhiran, K. Anti-oxidant and enzyme-inhibitory potential of marine Streptomyces. *Am. J. Biochem. Biotechnol.* **2013**, *9*, 282–290, doi:10.3844/ajbbsp.2013.282.290.

20. Jemimah Naine, S.; Nasimunislam, N.; Vaishnavi, B.; Mohanasrinivasan, V.; Subathra Devi, C. Isolation of soil actinomycetes inhabiting amrithi forest for the potential source of bioactive compounds. *Asian J. Pharm. Clin. Res.* **2012**, *5*, 189–192.

21. Gautham, S.A.; Onkarappa, R. In vitro antioxidant activity of metabolite from Streptomyces fradiae strain GOS1. *Int. J. Drug Dev. Res.* **2013**, *5*, 235–244.

22. Gautham, S.A.; Onkarappa, R.; Kuppast, I.J. Pharmacological activities of metabolite from Streptomyces fradiae strain GOS 1. *Int. J. Chem. Sci.* **2013**, *11*, 583–590.

23. Lee, S.K.; Lee, D.R.; Choi, B.K.; Palaniyandi, S.A.; Yang, S.H.; Suh, J.W. Glutathione S-transferase pi (GST-pi) inhibition and anti-inflammation activity of the ethyl acetate extract of Streptomyces sp. strain MJM 8637. *Saudi J. Biol. Sci.* **2015**, *22*, 744–751, doi:10.1016/j.sjbs.2015.04.003.

24. Saini, P.; Gangwar, M. Enzyme and free radical inhibitory potentials of ethyl acetate extract of endophytic actinomycete from Syzygium cumini. *Indian J. Biochem. Biophys.* **2017**, *54*, 207–213.

25. Akshatha, J. V; Prakash, H.S.; Nalini, M.S. Actinomycete Endophytes from the Ethno Medicinal Plants of Southern India: Antioxidant Activity and Characterization Studies. *J. Biol. Act. Prod. from Nat.* **2016**, *6*, 166–172, doi:10.1080/22311866.2016.1191971.

26. Nafis, A.; Kasrati, A.; Azmani, A.; Ouhdouch, Y.; Hassani, L. Endophytic actinobacteria of medicinal plant Aloe vera: Isolation, antimicrobial, antioxidant, cytotoxicity assays and taxonomic study. *Asian Pac. J. Trop. Biomed.* **2018**, *8*, 513–518, doi:10.4103/2221-1691.244160.

27. Wang, L.; Qiu, P.; Long, X.F.; Zhang, S.; Zeng, Z.G.; Tian, Y.Q. Comparative analysis of chemical constituents, antimicrobial and antioxidant activities of ethylacetate extracts of Polygonum cuspidatum and its endophytic actinomycete, Streptomyces sp. A0916. *Chin. J. Nat. Med.* **2016**, *14*, 117–123, doi:10.1016/S1875-5364(16)60004-3.

28. Axenov-Gribanov, D. V; Voytsekhovskaya, I. V; Rebets, Y. V; Tokovenko, B.T.; Penzina, T.A.; Gornostay, T.G.; Adelshin, R. V; Protasov, E.S.; Luzhetskyy, A.N.; Timofeyev, M.A. Actinobacteria possessing antimicrobial and antioxidant activities isolated from the pollen of scots pine (Pinus sylvestris) grown on the Baikal shore. *Antonie Van Leeuwenhoek* **2016**, *109*, 1307–1322, doi:10.1007/s10482-016-0730-5.

29. Nimal Christhudas, I.V.S.; Praveen Kumar, P.; Agastian, P. In Vitro α-Glucosidase Inhibition and Antioxidative Potential of an Endophyte Species (Streptomyces sp. Loyola UGC) Isolated from Datura stramonium L. *Curr. Microbiol.* **2013**, *67*, 69–76, doi:10.1007/s00284-013-0329-2.

30. Jasim, B.; Soumya, R.; Jyothis, M.; Radhakrishnan, E.K. Exploration of actinomycetes endophytically associated with Piper nigrum for potential bioactivity. *J. Microbiol. Biotechnol. Food Sci.* **2015**, *04*, 282–286, doi:10.15414/jmbfs.2015.4.4.282-286.

31. Tanvir, R.; Sajid, I.; Hasnain, S. Biotechnological potential of endophytic actinomycetes associated with Asteraceae plants: Isolation, biodiversity and bioactivities. *Biotechnol. Lett.* **2014**, *36*, 767–773, doi:10.1007/s10529-013-1430-0.

32. Khanam, W.; Vootla, S.K. Comparative study of Ocimum basillicum and its endophytic actinomycetes Streptomyces flavoviridis a3wk: evaluation of antioxidant, anti-inflammatory and antimicrobial activity. *Int. J. Pharm. Sci. Res.* **2018**, *9*, 1023–1034, doi:10.13040/IJPSR.0975-8232.9(3).1023-34.

33. Ser, H.L.; Tan, L.T.H.; Palanisamy, U.D.; Abd Malek, S.N.; Yin, W.F.; Chan, K.G.; Goh, B.H.; Lee, L.H. Streptomyces antioxidans sp. nov., a novel mangrove soil actinobacterium with antioxidative and neuroprotective potentials. *Front. Microbiol.* **2016**, *7*, 1–14, doi:10.3389/fmicb.2016.00899.

34. Veena, S.; Swetha, D.; Karthik, L.; Bhaskara Rao, K. V. Assessment of anti-typhoid and antioxidant activity of marine actinobacteria isolated from Chennai marine sediments. *Der Pharm. Lett.* **2016**, *8*, 166–172.

35. Subramanian, D.; Kim, M.S.; Kim, D.H.; Heo, M.S. Isolation, characterization, antioxidant, antimicrobial and cytotoxic effect of marine Actinomycete, Streptomyces Carpaticus MK-01, against fish pathogens. *Brazilian Arch. Biol. Technol.* **2017**, *60*, 1–9, doi:10.1590/1678-4324-2017160539.

36. Raghava Rao, K.V.; Raghava Rao, T. Molecular characterization and its antioxidant activity of a newly isolated Streptomyces coelicoflavus BC 01 from mangrove soil. *J. Young Pharm.* **2013**, *5*, 121–126, doi:10.1016/j.jyp.2013.10.002.

37. Siddharth, S.; Vittal, R. Evaluation of Antimicrobial, Enzyme Inhibitory, Antioxidant and Cytotoxic Activities of Partially Purified Volatile Metabolites of Marine Streptomyces sp.S2A. *Microorganisms* **2018**, *6*, 72, doi:10.3390/microorganisms6030072.

38. Singhania, M.; Ravichander, P.; Swaroop, S.; Naine Selvakumar, J.; Vaithilingam, M.; Devi Chandrasekaran, S. Anti-bacterial and anti-oxidant property of Streptomyces laurentii VITMPS isolated from marine soil. *Curr. Bioact. Compd.* **2016**, *13*, 78–81, doi:10.2174/1573407212666160606130704.

39. Ser, H.L.; Palanisamy, U.D.; Yin, W.F.; Chan, K.G.; Goh, B.H.; Lee, L.H. Streptomyces malaysiense sp. nov.: A novel Malaysian mangrove soil actinobacterium with antioxidative activity and cytotoxic potential against human cancer cell lines. *Sci. Rep.* **2016**, *6*, 1–12, doi:10.1038/srep24247.

40. Ser, H.L.; Palanisamy, U.D.; Yin, W.F.; Abd Malek, S.N.; Chan, K.G.; Goh, B.H.; Lee, L.H. Presence of antioxidative agent, Pyrrolo[1,2-a]pyrazine-1,4-dione, hexahydro- in newly isolated Streptomyces mangrovisoli sp. nov. *Front. Microbiol.* **2015**, *6*, 1–11, doi:10.3389/fmicb.2015.00854.

41. Law, J.W.F.; Ser, H.L.; Ab Mutalib, N.S.; Saokaew, S.; Duangjai, A.; Khan, T.M.; Chan, K.G.; Goh, B.H.; Lee, L.H. Streptomyces monashensis sp. nov., a novel mangrove soil actinobacterium from East Malaysia with antioxidative potential. *Sci. Rep.* **2019**, *9*, 1–18, doi:10.1038/s41598-019-39592-6.

42. Tangjitjaroenkun, J. Evaluation of antioxidant, antibacterial, and gas chromatography-mass spectrometry analysis of ethyl acetate extract of streptomyces omiyaensis SCH2. *Asian J. Pharm. Clin. Res.* **2018**, *11*, 271–276, doi:10.22159/ajpcr.2018.v11i7.25692.

43. Jemimah Naine, S.; Subathra Devi, C.; Mohanasrinivasan, V.; Vaishnavi, B. Antimicrobial, antioxidant and cytotoxic activity of marine Streptomyces parvulus VITJS11 crude extract. *Brazilian Arch. Biol. Technol.* **2015**, *58*, 198–207, doi:10.1590/S1516-8913201400173.

44. Ser, H.L.; Mutalib, N.S.A.; Yin, W.F.; Chan, K.G.; Goh, B.H.; Lee, L.H. Evaluation of antioxidative and cytotoxic activities of Streptomyces pluripotens MUSC 137 isolated from mangrove soil in Malaysia. *Front. Microbiol.* **2015**, *6*, 1–11, doi:10.3389/fmicb.2015.01398.

45. Selvakumar, J.; Chandrasekaran, S.; Vaithilingam, M. Bio prospecting of marine-derived Streptomyces spectabilis VITJS10 and exploring its cytotoxicity against human liver cancer cell lines. *Pharmacogn. Mag.* **2015**, *11*, 469, doi:10.4103/0973-1296.168974.

46. Mandal, S.; Divya; Sreedharan, V.; Rao, K.V.B. Bioactive potential of streptomyces variabilis - DV-35 isolated from thottada marine sediments, Kannur, Kerala. *Asian J. Pharm. Clin. Res.* **2016**, *9*, 67–71, doi:10.22159/ajpcr.2016.v9s3.14643.

47. Eva, S.M.; Baraka, S.; Ken, H.M.M. Cytotoxicity and antioxidant activity of a streptomyces sp. from mangrove sediments of dar es salaam, Tanzania. *Int. J. Pharm. Pharm. Sci.* **2014**, *6*, 563–566.

48. Karthik, L.; Kumar, G.; Rao, K.V.B. Antioxidant activity of newly discovered lineage of marine actinobacteria. *Asian Pac. J. Trop. Med.* **2013**, *6*, 325–332, doi:10.1016/S1995-7645(13)60065-6.

49. Tan, L.T.H.; Chan, K.G.; Khan, T.M.; Bukhari, S.I.; Saokaew, S.; Duangjai, A.; Pusparajah, P.; Lee, L.H.; Goh, B.H. Streptomyces sp. MUM212 as a source of antioxidants with radical scavenging and metal chelating properties. *Front. Pharmacol.* **2017**, *8*, 1–18, doi:10.3389/fphar.2017.00276.

50. Tan, L.T.H.; Ser, H.L.; Yin, W.F.; Chan, K.G.; Lee, L.H.; Goh, B.H. Investigation of antioxidative and anticancer potentials of Streptomyces sp. MUM256 isolated from Malaysia mangrove soil. *Front. Microbiol.* **2015**, *6*, doi:10.3389/fmicb.2015.01316.

51. Tan, L.T.H.; Chan, K.G.; Pusparajah, P.; Yin, W.F.; Khan, T.M.; Lee, L.H.; Goh, B.H. Mangrove derived Streptomyces sp. MUM265 as a potential source of antioxidant and anticolon-cancer agents. *BMC Microbiol.* **2019**, *19*, 1–16, doi:10.1186/s12866-019-1409-7.

52. Tan, L.T.H.; Chan, K.G.; Chan, C.K.; Khan, T.M.; Lee, L.H.; Goh, B.H. Antioxidative potential of a streptomyces sp. MUM292 isolated from mangrove soil. *Biomed Res. Int.* **2018**, *2018*, doi:10.1155/2018/4823126.

53. Kamala, K.; Karuppiah, V.; Sivakumar, K. Comparative evaluation of in vitro antioxidant potent of the marine actinobacteria from gulf of Mannar Biosphere Reserve. *Int. J. Pharma Bio Sci.* **2013**, *4*, 207–216.

54. Sengupta, S.; Pramanik, A.; Ghosh, A.; Bhattacharyya, M. Antimicrobial activities of actinomycetes isolated from unexplored regions of Sundarbans mangrove ecosystem. *BMC Microbiol.* **2015**, *15*, 170/1-170/16, doi:10.1186/s12866-015-0495-4.

55. Almasi, F.; Mohammadipanah, F.; Adhami, H.R.; Hamedi, J. Introduction of marine-derived Streptomyces sp. UTMC 1334 as a source of pyrrole derivatives with anti-acetylcholinesterase activity. *J. Appl. Microbiol.* **2018**, *125*, 1370–1382, doi:10.1111/jam.14043.

56. Jemimah Naine, S.; Subathra Devi, C.; Mohanasrinivasan, V.; George Priya Doss, C. Bioactivity of Marine Streptomyces sp. VITJS4: Interactions of Cytotoxic Phthalate Derivatives with Human Topoisomerase II α: An In Silico Molecular Docking Analysis. *Interdiscip. Sci. Comput. Life Sci.* **2018**, *10*, 261–270, doi:10.1007/s12539-016-0187-2.

57. Duraikannu, D.; D. Chandrasekaran, S.; N. Selvakumar, J.; Vaithilingam, M. A Preliminary Study of In vitro Antioxidant and Antibacterial Activity of Streptomyces gancidicus VITSD1 Isolated from Marine Soil. *Curr. Bioact. Compd.* **2014**, *10*, 292–297, doi:10.2174/1573407211666141216195604.

58. Sanjivkumar, M.; Babu, D.R.; Suganya, A.M.; Silambarasan, T.; Balagurunathan, R.; Immanuel, G. Investigation on pharmacological activities of secondary metabolite extracted from a mangrove associated actinobacterium Streptomyces olivaceus (MSU3). *Biocatal. Agric. Biotechnol.* **2016**, *6*, 82–90, doi:10.1016/j.bcab.2016.03.001.

59. Pooja, S.; Aditi, T.; Naine, S.J.; Subathra Devi, C. Bioactive compounds from marine Streptomyces sp. VITPSA as therapeutics. *Front. Biol. (Beijing).* **2017**, *12*, 280–289, doi:10.1007/s11515-017-1459-x.

60. El-Shenawy, N.S. Effect of Streptomyces 23-2B metabolites on hepatic lipid peroxidation and some antioxidant parameters in Wister rats. *World J. Microbiol. Biotechnol.* **2010**, *26*, 2185–2191, doi:10.1007/s11274-010-0403-x.

61. Gozari, M.; Bahador, N.; Jassbi, A.R.; Mortazavi, M.S.; Eftekhar, E. Antioxidant and cytotoxic activities of metabolites produced by a new marine Streptomyces sp. isolated from the sea cucumber Holothuria leucospilota. *Iran. J. Fish. Sci.* **2018**, *17*, 413–426, doi:10.22092/IJFS.2018.116076.

62. Lertcanawanichakul, M.; Chawawisit, K.; Pondet, K.; Kwantep, J. Quantitation of total phenolic contents of bioactive compounds fractions Streptomyces species. *Int. J. PharmTech Res.* **2015**, *7*, 320–324.

63. Latha, Y.; Vasavithirumalanadhuni; Mathakala, V.; Devi, P.U.M. In vivo anti-inflammatory activity of ethyl acetate extract derived from marine Streptomyces carpaticus. *Int. J. Pharm. Sci. Res.* **2017**, *8*, 5221–5226, doi:10.13040/IJPSR.0975-8232.8(12).5221-26.
