# Supplementary material for: Streptomyces-Derived Metabolites with Potential Photoprotective Properties—A Systematic Literature Review and Meta-Analysis on the Reported Chemodiversity
Source: Molecules. 2020 Jul 15;25(14):3221. doi: 10.3390/molecules25143221 (PMC7397340; doi:10.3390/molecules25143221)
Supplement: Supplementary file 1 [file molecules-25-03221-s001.zip › Supplementary Materials/Table_S5.docx]

**Table S5.** Databases search results.

| **Database** | **Date** | **Keywords** | **Hits** |
| --- | --- | --- | --- |
| Scopus | 21/05/2019 | streptomyces AND anti-inflammatory OR antioxidant | 378 |
| Web of Science | 21/05/2019 | streptomyces AND (anti-inflammatory OR antioxidant) | 316 |
| PubMed | 21/05/2019 | streptomyces AND (anti-inflammatory OR antioxidant) | 654 |
| Scielo | 21/05/2019 | streptomyces AND (anti-inflammatory OR antioxidant) | 3 |
| Sage Journals | 21/05/2019 | streptomyces AND (anti-inflammatory OR antioxidant) | 141 |
| EBSCOhost Research | 21/05/2019 | streptomyces AND (anti-inflammatory OR antioxidant) | 228 |
| Scopus | 21/05/2019 | streptomyces AND "anti UV" OR photoaging OR photoprotection OR “photo protection” OR "sun protection" OR "sunlight protection" OR sunscreen OR sunscreening OR "UV protection" OR "ultraviolet blocking" OR "ultraviolet shielding" OR “UV absorbing” | 19 |
| Web of Science | 21/05/2019 | streptomyces AND ("anti UV" OR photoaging OR photoprotection OR "photo protection" OR "sun protection" OR "sunlight protection" OR sunscreen OR sunscreening OR "UV protection" OR "ultraviolet blocking" OR "ultraviolet shielding" OR "UV absorbing") NOT cyanobacteria | 12 |
| PubMed | 21/05/2019 | streptomyces AND ("anti UV" OR photoaging OR photoprotection OR "photo protection" OR "sun protection" OR "sunlight protection" OR sunscreen OR sunscreening OR "UV protection" OR "ultraviolet blocking" OR "ultraviolet shielding" OR "UV absorbing") NOT cyanobacteria | 10 |
| Scielo | 21/05/2019 | streptomyces AND ("anti UV" OR photoaging OR photoprotection OR "photo protection" OR "sun protection" OR "sunlight protection" OR sunscreen OR sunscreening OR "UV protection" OR "ultraviolet blocking" OR "ultraviolet shielding" OR "UV absorbing") NOT cyanobacteria | 1 |
| Sage Journals | 21/05/2019 | streptomyces AND ("anti UV" OR photoaging OR photoprotection OR "photo protection" OR "sun protection" OR "sunlight protection" OR sunscreen OR sunscreening OR "UV protection" OR "ultraviolet blocking" OR "ultraviolet shielding" OR "UV absorbing") NOT cyanobacteria | 17 |
| EBSCOhost Research | 21/05/2019 | streptomyces AND ("anti UV" OR photoaging OR photoprotection OR "photo protection" OR "sun protection" OR "sunlight protection" OR sunscreen OR sunscreening OR "UV protection" OR "ultraviolet blocking" OR "ultraviolet shielding" OR "UV absorbing") NOT cyanobacteria | 10 |
| Total | | | 1789 |
