# Supplementary figures and images for: Streptomyces-Derived Metabolites with Potential Photoprotective Properties—A Systematic Literature Review and Meta-Analysis on the Reported Chemodiversity
Source: Molecules. 2020 Jul 15;25(14):3221. doi: 10.3390/molecules25143221 (PMC7397340; doi:10.3390/molecules25143221)

Table S4. Chemical structures of retrieved compounds.

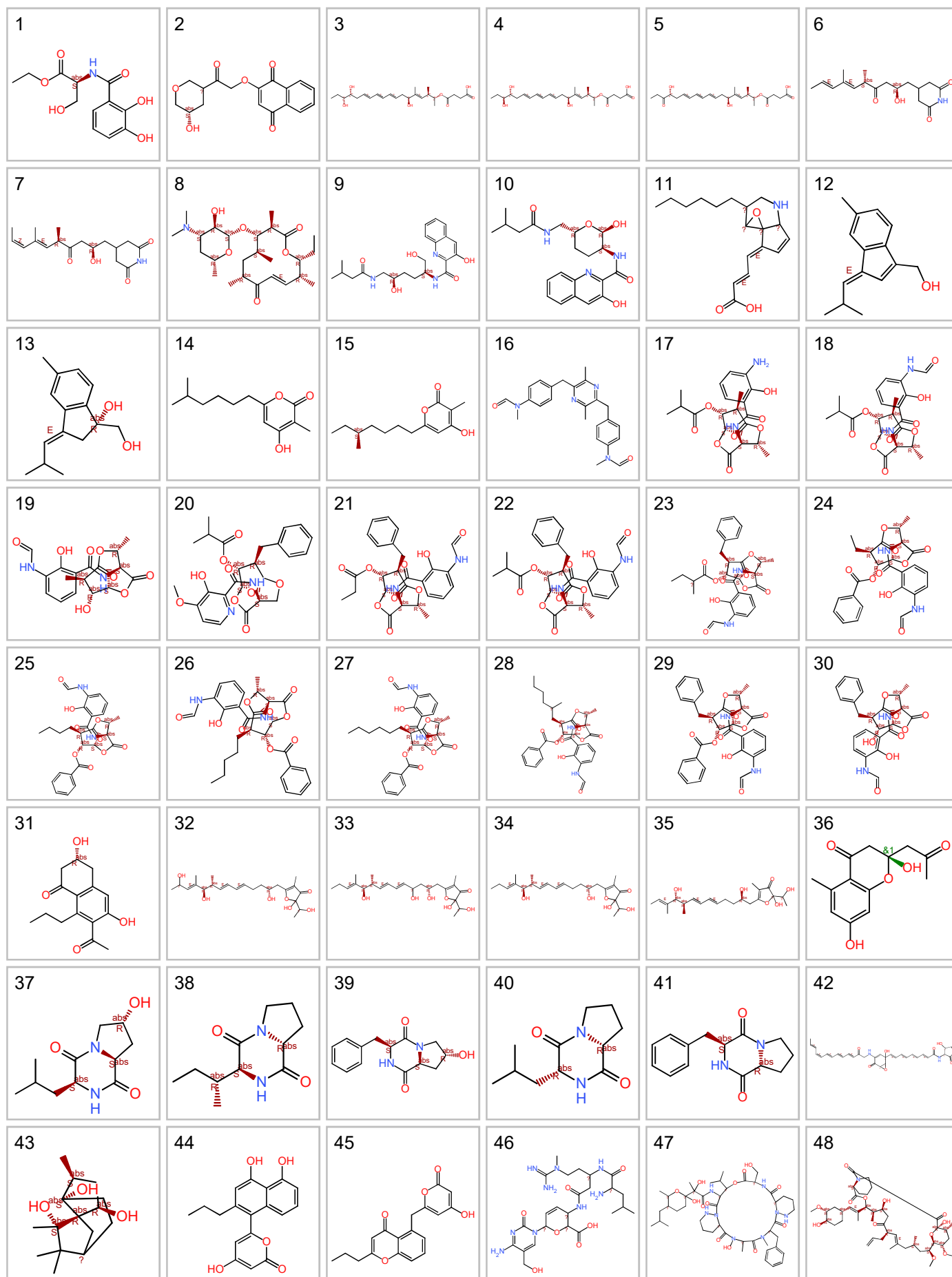

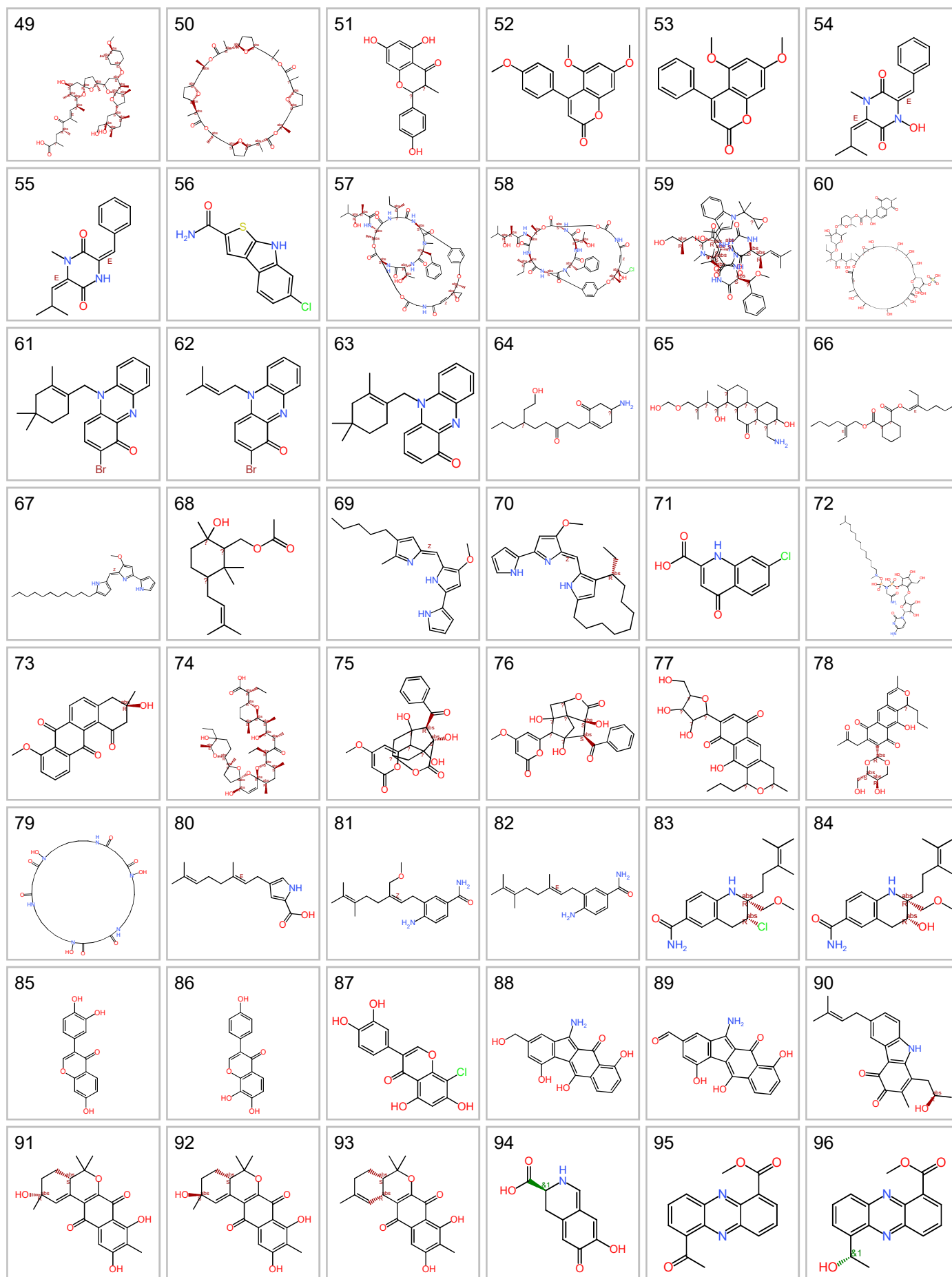

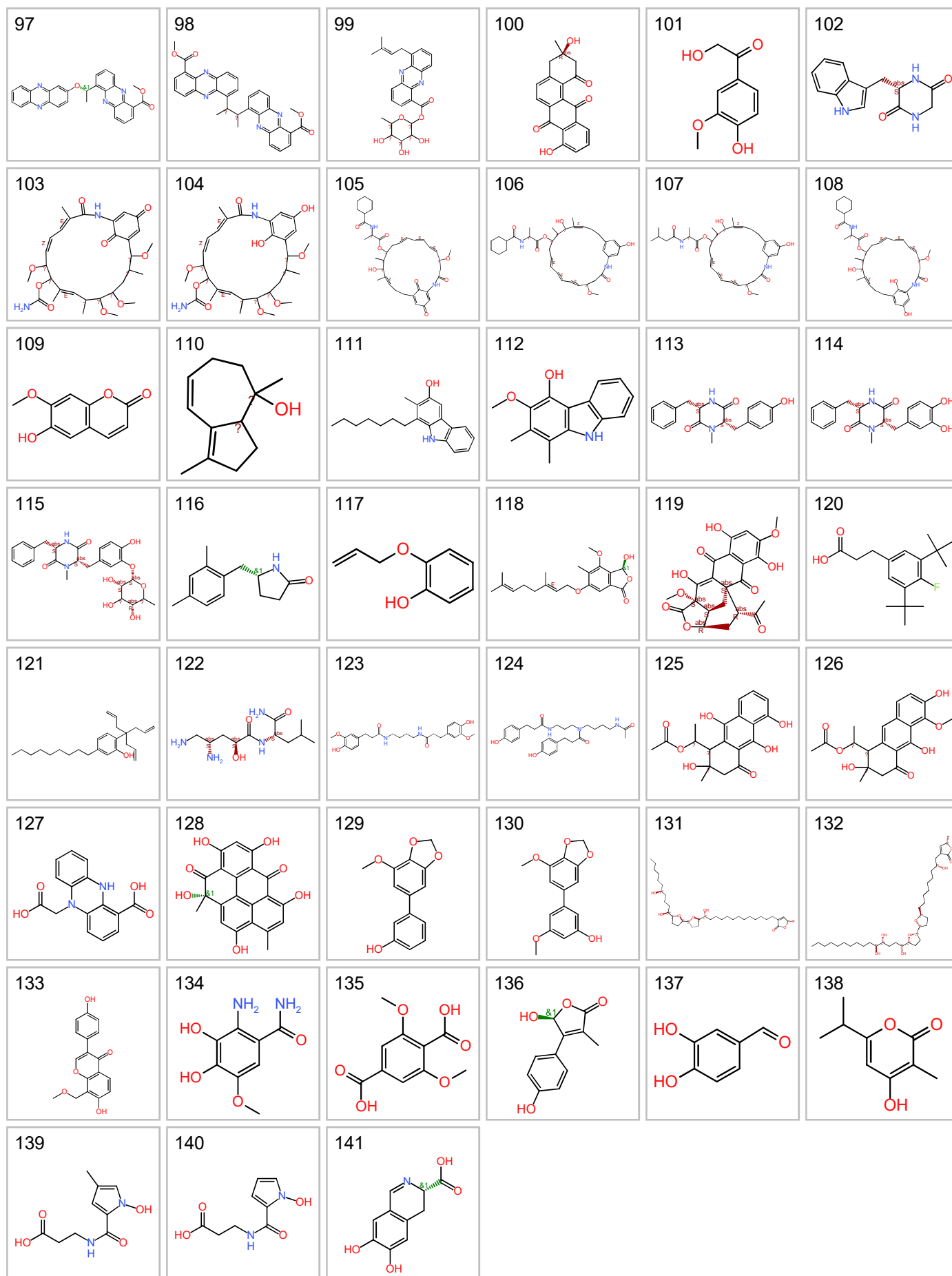

Supplement: Supplementary file 1 [file molecules-25-03221-s001.zip › Supplementary Materials/Table_S4.pdf]
